# Identification of three capsule depolymerases in a bacteriophage infecting *Klebsiella pneumoniae* capsular types K7, K20, and K27 and therapeutic application

**DOI:** 10.1186/s12929-023-00928-0

**Published:** 2023-05-20

**Authors:** Jia-Wen Wu, Jin-Town Wang, Tzu-Lung Lin, Ya-Zhu Liu, Lii-Tzu Wu, Yi-Jiun Pan

**Affiliations:** 1 Graduate Institute of Biomedical Sciences, College of Medicine, China Medical University, Taichung, Taiwan; 2 Department of Microbiology and Immunology, School of Medicine, College of Medicine, China Medical University, No. 91 Hsueh-Shih Road, Taichung, Taiwan; 3 Department of Microbiology, National Taiwan University College of Medicine, Taipei, Taiwan; 4 Department of Internal Medicine, National Taiwan University Hospital, Taipei, Taiwan; 5 Department of Medical Biotechnology and Laboratory Science, Chang Gung University, Taoyuan, Taiwan

**Keywords:** *Klebsiella pneumoniae*, Hypervirulent, Phage, Capsule depolymerase

## Abstract

**Background:**

*Klebsiella pneumoniae* capsular types K1, K2, K5, K20, K54, and K57 are prevalent hypervirulent types associated with community infections, and worrisomely, hypervirulent strains that acquired drug resistance have been found. In the search for alternative therapeutics, studies have been conducted on phages that infect *K. pneumoniae* K1, K2, K5, and K57-type strains and their phage-encoded depolymerases. However, phages targeting *K. pneumoniae* K20-type strains and capsule depolymerases capable of digesting K20-type capsules have rarely been reported. In this study, we characterized a phage that can infect *K. pneumoniae* K20-type strains, phage vB_KpnM‐20.

**Methods:**

A phage was isolated from sewage water in Taipei, Taiwan, its genome was analyzed, and its predicted capsule depolymerases were expressed and purified. The host specificity and capsule-digesting activity of the capsule depolymerases were determined. The therapeutic effect of the depolymerase targeting *K. pneumoniae* K20-type strains was analyzed in a mouse infection model.

**Results:**

The isolated *Klebsiella* phage, vB_KpnM‐20, infects *K. pneumoniae* K7, K20, and K27-type strains. Three capsule depolymerases, K7dep, K20dep, and K27dep, encoded by the phage were specific to K7, K20, and K27-type capsules, respectively. K20dep also recognized *Escherichia coli* K30-type capsule, which is highly similar to *K. pneumoniae* K20-type. The survival of *K. pneumoniae* K20-type-infected mice was increased following administration of K20dep.

**Conclusions:**

The potential of capsule depolymerase K20dep for the treatment of *K. pneumoniae* infections was revealed using an in vivo infection model. In addition, K7dep, K20dep, and K27dep capsule depolymerases could be used for *K. pneumoniae* capsular typing.

**Supplementary Information:**

The online version contains supplementary material available at 10.1186/s12929-023-00928-0.

## Background

*Klebsiella pneumoniae*, an opportunistic pathogen, is associated with various nosocomial and community-acquired infections, including soft tissue infections, urinary tract infections, surgical site infections, catheter-related infections, pneumonia, and septicemia [1, 2]. Since the mid-1980s, community-acquired pyogenic liver abscesses, complicated by metastatic meningitis and endophthalmitis, caused by *K. pneumoniae* have emerged globally [3–9]. Besides, the rapid spread of carbapenem and multi-drug resistance in *K. pneumoniae* is of great concern [10–14]. Colistin was reintroduced into the clinical setting to eradicate carbapenem-resistant *K. pneumoniae* (CRKP) [15, 16]. However, colistin-resistant CRKP strains have been reported, at a rate of ~ 40% in some countries [17–19]. In 2017, the World Health Organization named CRKP as priority bacteria for urgent development of new antimicrobials [20].

Growing resistance to several classes of antibiotics has renewed interest in alternative treatments, such as phage therapy, for controlling hard-to-treat bacterial infections. Several promising *K. pneumoniae* phages have been reported, and their potential therapeutic efficacy has been demonstrated using infection models in *Galleria mellonella* larvae and mice [21–25]. Bacteriophage-encoded antibacterial proteins, such as capsule depolymerases, have also shown promise against various Gram-negative bacteria [26–28].

The capsule is a major virulence factor in *K. pneumoniae* that provides protection against phagocytosis and the bactericidal effects of serum; thus, enzymes with capsule-digesting activity have antimicrobial potential. Over 130 *Klebsiella* capsular types (K-types) have been described, and the clinical importance of different K-types has been investigated. Six K-types, K1, K2, K5, K20, K54, and K57, are thought to be closely related to hypervirulence and associated with community invasive disease and pyogenic liver abscesses in Taiwan [29–33], where these K-types also account for ~ 70% of *K. pneumoniae*-caused community-acquired pneumonia [34]. K1, K2, and K20-types were reported to be closely associated with hypermucoviscosity—an indicator of hypervirulence [35]. One study in China analyzed the K-types of 21 hypermucoviscous CRKP strains; ~ 33% were K20, and ~ 5% were K2 [36]. Another study in Iran identified K20 as the prevalent (~ 55%) hypervirulent K-type [37]. Many of these strains carried *bla*_NDM-1_/*bla*_OXA-48_, suggesting that K20-type may be associated with carbapenem resistance and hypermucoviscosity*.*

Several phages infecting K1, K2, K5, and K57-type strains and their depolymerases have been characterized [23, 24, 38, 39]. However, to our knowledge, phages infecting K20-type *K. pneumoniae* and K20-type-specific capsule depolymerases have rarely been reported. Here, we isolated a phage that infects *K. pneumoniae* K7, K20, and K27-type strains and characterized its three K-type-specific depolymerases. One of the depolymerases, K20dep, was further analyzed for its efficacy in treating K20-type-infected mice. The results suggest phage-encoded capsule depolymerases as a potential alternative for infection control. These capsule depolymerases may also be useful tools for capsular typing.

## Methods

### Bacterial strains

One *Escherichia coli* strain, O9:K30:H12 (Statens Serum Institut, Copenhagen, Denmark), and 95 *Klebsiella* strains (representing 82 K-types) were used for host range determination. The *Klebsiella* strains included 76 K-type reference strains (Statens Serum Institut), 1 strain showing K1-type (NTUH-K2044), 2 strains showing K20-type (A13 and 5262), and 16 strains showing KN1, KN2, KN3, KN4, or KN5-type (Additional file 1: Table S1). Two K20-type strains, A13 and KP440 (a clinical CRKP strain), were used in the mouse study.

### Phage isolation

Sewage water (4 ml) collected in Taipei was mixed with 0.5 ml of a freshly cultured *Klebsiella* K27-type reference strain (K27ref) and cultured with 1 ml of 5X Luria–Bertani (LB) medium (Bioshop, Burlington, Canada) at 37 °C overnight. After centrifugation, the supernatant was filtered (0.45 μm filter; Merck Millipore, Tullagreen, Germany) and spotted on LB plates overlaid with strain K27ref to detect phage plaques. The agar overlay method was used to isolate pure phage and determine the titer [40].

### Determination of host range and capsule depolymerase activity

To determine which bacterial strains were permissive to phage infection and the activity range of the capsule depolymerases, spot tests were conducted [41]. An LB agar plate was overlaid with top agar containing 200 μl of fresh bacterial culture. Aliquots of phage containing 10^8^ plaque-forming units or various amounts of purified recombinant capsule depolymerase (100, 50, 40, 30, 20, 10, 5, 2.5, and 1 ng/4 μl) were spotted onto the plate. After overnight incubation at 37 °C, lytic or semi-clear spots were observed.

### Efficiency of plating assay

The ability of the phage to infect different hosts was quantified using a previously described efficiency of plating (EOP) method [42]. Briefly, tenfold serial dilutions of phage suspensions (1 × 10^1^–1 × 10^9^/ml) were prepared with SM buffer (100 mM NaCl, 8 mM MgSO_4_·7H_2_O, and 50 mM Tris, pH 7.5). For plating, 100 μl of an exponential phase culture of the test strain (1 × 10^8^ colony-forming units (CFU)/ml) was mixed with 100 μl of a phage dilution and incubated for 10 min. Then, 4 ml of 0.7% top agar was added, and the mixture was poured onto LB agar, incubated at 37 °C for 18 h, and scored for the presence of plaques. The EOP for each strain was obtained by comparing to the EOP for strain K27ref, which showed the highest efficiency and was set to 100% (control strain), and three independent experiments were analyzed.

### Transmission electron microscopy

A phage suspension was layered on top of a CsCl step gradient (densities: 1.1 and 1.7 g/ml; Sigma-Aldrich) and centrifuged in a SW41 Ti swinging bucket rotor at 66,000 × *g* for 16 h at 4 °C. Phage was collected from the visible hazy blue/white bands using a syringe with a 23 G needle, and CsCl was removed by buffer exchange in ddH_2_O using an Amicon Ultra centrifugal filter (100,000 MWCO; Millipore). Purified phage was applied to carbon-coated nitrocellulose grids, stained with 2% uranyl acetate, and examined using a Hitachi H-7100 transmission electron microscope.

### Genome sequence analysis

Phage genomic DNA was isolated using the Qiagen Lambda kit (Qiagen, Valencia, CA, United States). Sequencing was performed using the Illumina/Solexa GAII sequencing platform with the High-throughput Genome Analysis Core at Yang-Ming Genome Research Center. Phage DNA (50 ng) was used to construct a sequencing library using the Illumina-compatible Nextera DNA Sample Prep Kit (Epicenter Biotechnologies, Madison, WI, United States) according to the manufacturer’s instructions. The library was quantified through quantitative PCR, and the library size was determined using a 2100 Bio analyzer (Agilent Technologies, Santa Clara, CA, United States) with a high-sensitivity DNA chip. Sequencing was performed on an Illumina HiSeq2000 system through paired-end sequencing (100 bp read length). The sequencing reads were trimmed to remove low quality sequences (< Q20) and adapters and assembled de novo using the CLC Genomics Workbench (CLC bio, Denmark). Gaps were filled using Sanger sequencing. The termini of the phage genome were investigated through PCR with primers (Additional file 1: Table S2) located at the end of genome sequences obtained from the whole-genome sequencing described above. The products were subjected to Sanger sequencing, which showed a circular map of the genome. The phage genome and coding region were analyzed using NCBI nucleotide BLAST and visualized using Easyfig [43]. Coding sequences were predicted using Vector NTI and annotated using NCBI protein BLAST. Sequences encoding capsule depolymerase were further analyzed using NCBI Conserved Domain Database and Multiple Sequence Alignment Viewer, GenomeNet MOTIF Search (MotifFinder) against Pfam library, and HHpred [44–46].

### Extraction and quantification of exopolysaccharide

The exopolysaccharide (EPS) of bacteria was extracted as previously described [47]. Briefly, a 500 μl overnight bacterial culture (OD_595_ = 1) was mixed with 100 μl of 1% Zwittergent 3–14 (Sigma-Aldrich) in 100 mM citric acid (pH 2.0). After 20 min at 50 °C, the mixture was centrifuged at 17,700 × *g* for 10 min. A 250 μl aliquot of the supernatant was transferred to a clean tube and precipitated with 1 ml of ethanol at 4 °C for 20 min. After centrifugation, the pellet was dried and dissolved in ddH_2_O. The sample was appropriately diluted, and then 1,200 μl of 12.5 mM sodium tetraborate (Sigma-Aldrich) in H_2_SO_4_ was added. The mixture was boiled for 5 min. After cooling, 20 μl of 0.15% 3-hydroxydiphenol (Sigma-Aldrich) was added, and the absorbance at 520 nm was measured. A standard curve ranging from 0.9375 to 30 μg of D-glucuronic acid (Sigma-Aldrich) dissolved in 200 μl of ddH_2_O was used to calculate the concentration of the diluted EPS [48]. The extracted EPS from 3 ml of the tested strains ranged from ~ 70 to ~ 200 μg (different in each strain) in 100 μl of ddH_2_O.

### Alcian blue staining

Extracted EPS (8 μg) was incubated with purified capsule depolymerase (3 μg) in 15 μl of reaction volume at 37 °C for 1, 4, or 24 h and then visualized by 10% SDS-PAGE. The gel was stained with Alcian blue (Sigma-Aldrich) as previously described [49, 50]. Briefly, the gel was washed three times (for 5, 10, and 15 min) with fix/wash solution (25% ethanol and 10% acetic acid) at 50 °C, soaked with 0.125% Alcian blue in fix/wash solution for 15 min at 50 °C, and then destained with fix/wash solution. EPS was visualized as blue-stained material.

### Mouse infection model

Twenty 5-week-old female BALB/cByl mice were intraperitoneally inoculated with ~ 3 × 10^3^ CFU of *K. pneumoniae* strain A13 (hypervirulent strain with low median lethal dose (LD_50_) of < 1 × 10^3^ CFU), and another 20 mice were inoculated with ~ 3 × 10^7^ CFU of *K. pneumoniae* strain KP440 (carbapenem-resistant strain with high LD_50_ of ~ 1 × 10^6^ CFU). The mice infected with each strain were then assigned to five groups (n = 4/group): control (1 group), treatment with elution buffer (EB; 2 groups: 1 and 8 h), and treatment with K20dep (2 groups: 1 and 8 h). At 1 or 8 h after bacterial infection, mice in the groups except control were injected intraperitoneally with 100 μl of either EB only or EB containing purified K20dep protein (35 µg).

### Statistical analysis

Phage EOP on different strains was analyzed with a paired* t*-test from three independent experiments. Mouse survival during a 30-day observation period was visualized using Kaplan–Meier analysis with a log-rank test [24]. All statistical analyses were conducted using GraphPad Prism 5.01, and a p-value < 0.05 was considered significant.

## Results

### Bacteriophage vB_KpnM‐20 infects *Klebsiella* K7, K20, and K27-type strains

**Fig. 1:**
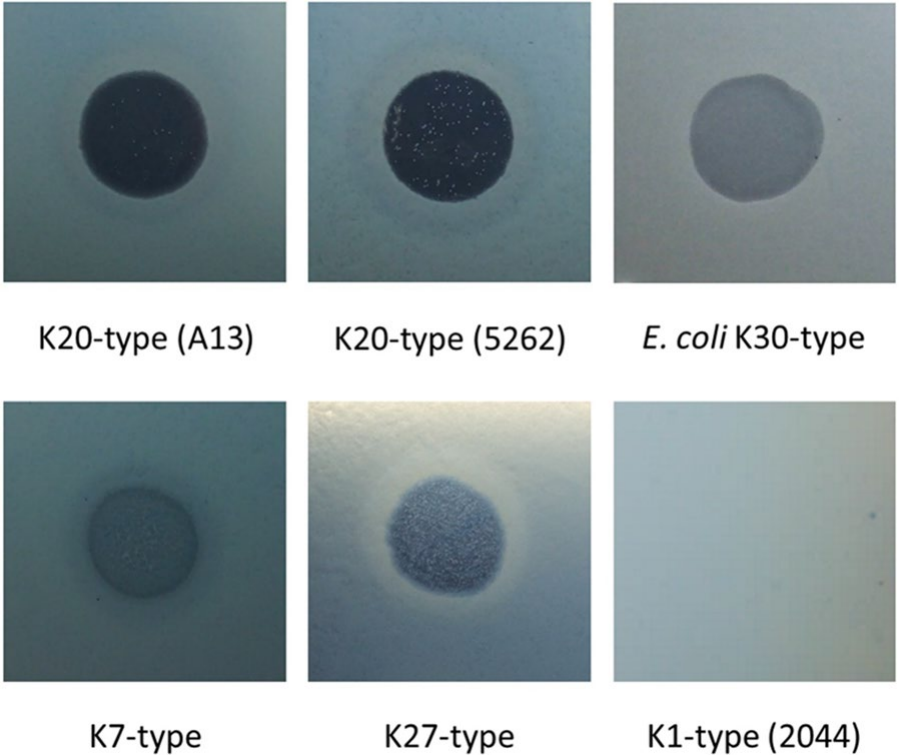
Spot tests of phage vB_KpnM‐20. Aliquots containing 10^8^ plaque-forming units of phage vB_KpnM‐20 were spotted on individual lawns of *Klebsiella* K7 (K7ref), K20 (A13 and 5262), K27 (K27ref), and K1-type (NTUH-K2044) strains and an *Escherichia coli* K30-type strain

**Fig. 2:**
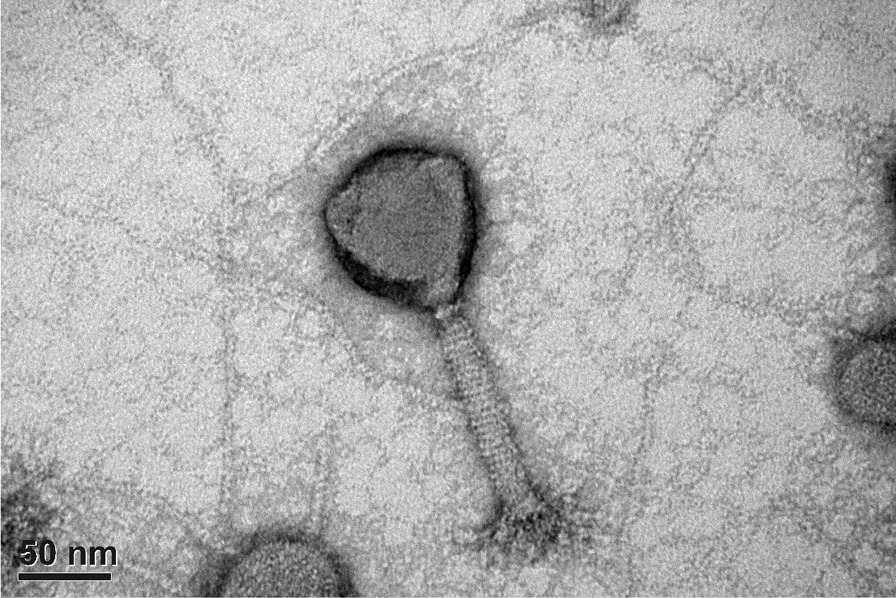
Electron micrograph of phage vB_KpnM‐20. Purified phage vB_KpnM‐20 particles showed icosahedral heads and contractile tails

A bacteriophage isolated from sewage water was named vB_KpnM‐20, and its host range was determined by spotting on lawns of 95 *Klebsiella* strains (82 K-types) (Additional file 1: Table S1). Phage vB_KpnM‐20 formed lytic spots on *Klebsiella* K7 (K7ref), K20 (A13 and 5262), and K27-type (K27ref) strains but did not form lytic spots on the other tested strains showing different K-types, including K1-type (NTUH-K2044), suggesting that this phage is specific to three *Klebsiella* K-types (Fig. 1 and Additional file 2: Fig. S1a). Phage vB_KpnM‐20 also infected an *E. coli* K30-type strain, which has a capsule that is highly similar to *Klebsiella* K20-type (Fig. 1 and Additional file 2: Fig. S1a) [51]. The plaques formed by phage vB_KpnM‐20 on *K. pneumoniae* K7, K20, and K27-type strains were ~ 1 mm diameter, clear, and surrounded by halos, suggesting that the phage might carry capsule depolymerases that digest these three K-types. The purified phage particles were visualized using the transmission electron microscope, which showed myovirus-like morphology—icosahedral heads and contractile tails (Fig. 2). From eight visualized phage particles, the average head diameter was 85 nm, and the average tail length was 136 nm.

### Infectivity of phage vB_KpnM‐20 varies in different hosts

**Fig. 3:**
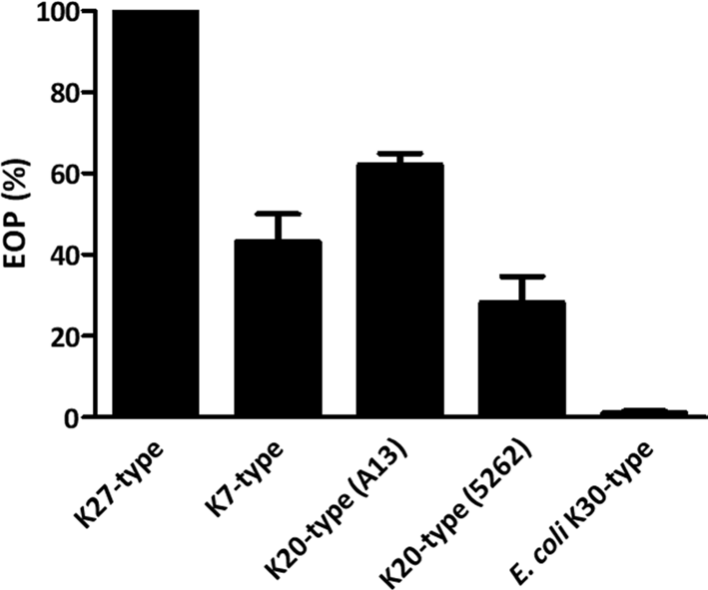
Infectivity of phage vB_KpnM‐20 in different strains. An efficiency-of-plating (EOP) assay was performed to quantitate phage infectivity in different hosts. The titer of phage vB_KpnM‐20 was determined using *Klebsiella* strain K27ref on which EOP was set to 100% (control). The EOP for each strain was obtained by comparing to the EOP for strain K27ref. For example, the EOP for strain A13 was 61.3% (65/106) compared to strain K27ref (Additional file 2: Fig. S1b). The EOP values from three independent experiments were compared and analyzed

An EOP assay was used to quantify phage infectivity in different strains (Fig. 3 and Additional file 2: Fig. S1b). Among the four *Klebsiella* strains tested, phage vB_KpnM‐20 showed the highest infectivity toward strain K27ref (control). Interestingly, distinct infectivity was observed in the K20-type strains and the *E. coli* K30-type strain, which express highly similar capsules (A13 and 5262, p = 0.0251; A13 and *E. coli* K30-type strain, p = 0.0017; 5262 and *E. coli* K30-type strain, p = 0.0460). A 50-fold difference was even observed between A13 and the *E. coli* K30-type strain.

### Analysis of the genome sequence of phage vB_KpnM‐20

**Fig. 4:**
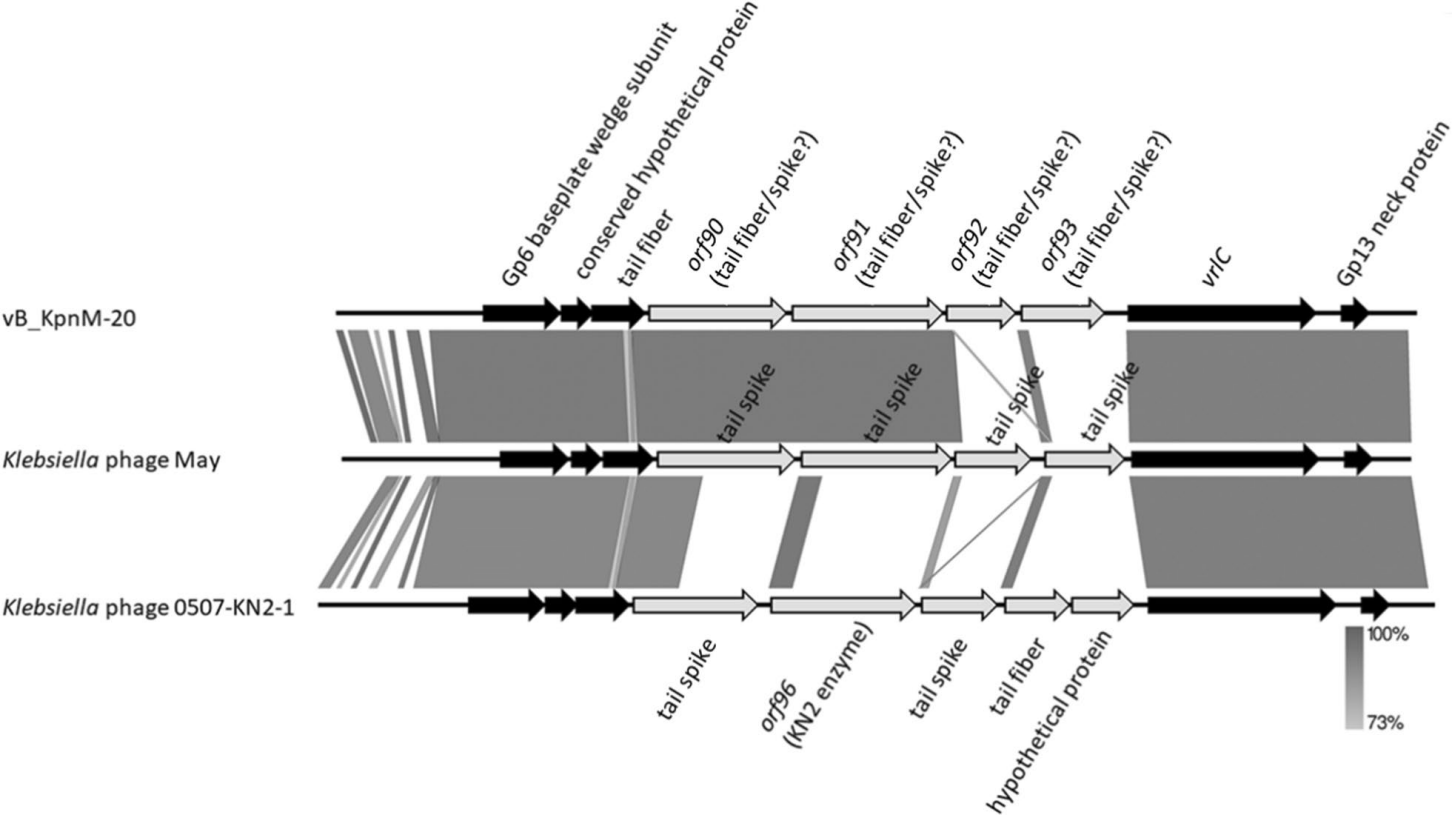
Genomic comparisons of phage vB_KpnM‐20, phage May, and phage 0507-KN2-1. Partial genome sequences neighboring the variable region (n.t.108307–120568) flanked by conserved tail fiber and *vrlC* genes of phage vB_KpnM‐20 and similar phages were compared using Easyfig. Genes located in the variable region are shown as gray arrows, while the conserved upstream and downstream genes are shown in black

Sequencing showed that the phage vB_KpnM‐20 genome is 158,784 bp in length (accession number MZ826764), with 46.5% G + C content and 125 open reading frames (> 500 bp in length). Phage vB_KpnM‐20 shares high sequence similarity with *Klebsiella* phage 0507-KN2-1 (accession number AB797215.1; 87% coverage, 98.34% identity), which has a circular genome, and *Klebsiella* phage May (accession number MG428991.1; 90% coverage, 93.97% identity) [52]. Phage 0507-KN2-1 is a KN2-type-specific phage carrying a KN2-type-specific enzyme, ORF96 [52]. The KN2-type depolymerase is located in a region (flanked by conserved tail fiber and *vrlC* genes) with low sequence similarity to vB_KpnM‐20 (Fig. 4). In this variable region in phage vB_KpnM‐20 (n.t.108307–120568), four genes, *orf90* (n.t.108361–111903; 1180 amino acids), *orf91* (n.t.112008–115892; 1294 amino acids), *orf92* (n.t.115936–117744; 602 amino acids), and *orf93* (n.t.117838–120009; 723 amino acids), were found. The encoded protein ORF90 is similar to a tail protein of phage May (accession number YP_009796159.1; 100% coverage, 99.58% identity). ORF91 is similar to a tail fiber protein of phage May (accession number YP_009796160.1; 99% coverage, 97.75% identity). ORF92 is similar to a hypothetical protein of *Klebsiella* phage K751 (accession number UPW36206.1; 83% coverage, 88.91% identity) and a phage tail fiber protein in *K. pneumoniae* (accession number WP_181502371.1; 86% coverage, 72.83% identity). ORF93 is similar to another hypothetical protein of phage K751 (accession number UPW36008.1; 100% coverage, 99.31% identity). Sequences of two groups of tail fiber/spike proteins, for example, a non-contractile tail fiber protein of *Klebsiella* phage vB_KpnS-VAC70 (accession number UEW68236.1; 84% coverage, 67.59% identity) and a tail spike protein of *Klebsiella* phage vB_KpnM_KpS110 (accession number YP_009798897.1; 11% coverage, 84.88% identity), can be aligned to different parts of ORF93 (residue 114–722 and 1–86, respectively).

Highly conserved amino acid positions were found in the alignments of ORF90 (~ residue 1–410) with similar sequences mostly from tail proteins, tail fibers/spikes, endo-N-neuraminidases, and hypothetical proteins in the database. Similar results of conservation were detected in ORF91 (~ residue 220–1290) and ORF92 (~ residue 120–240). Conserved peptidase of C-terminal intramolecular chaperone domain was identified in ORF90 (residue 1023–1171; E-value = 4.85e−29) and ORF91 (residue 1125–1278; E-value = 1.89e−33); one catalytic domain of bacteriophage endosialidase from the Pfam database was found in ORF91 (residue 1079–1124; E-value = 0.054). The analysis using HHpred, a server for remote protein homology detection, also indicated the homology of the four proteins to tail spike proteins in certain regions. The above bioinformatic analyses are shown in the Additional files (﻿Additional file 3: results shown using the Multiple Sequence Alignment viewer, Additional file 1: Table S3, and Additional file 2: Fig. S2).

Above all, *orf90*, *orf91*, *orf92*, and *orf93* showed high possibility of encoding tail fiber/spike proteins. Since phage tail fiber/spike proteins were commonly found showing capsule depolymerase activities, we expressed the four genes of phage vB_KpnM‐20.

### Expression of the putative capsule depolymerases

**Fig. 5:**
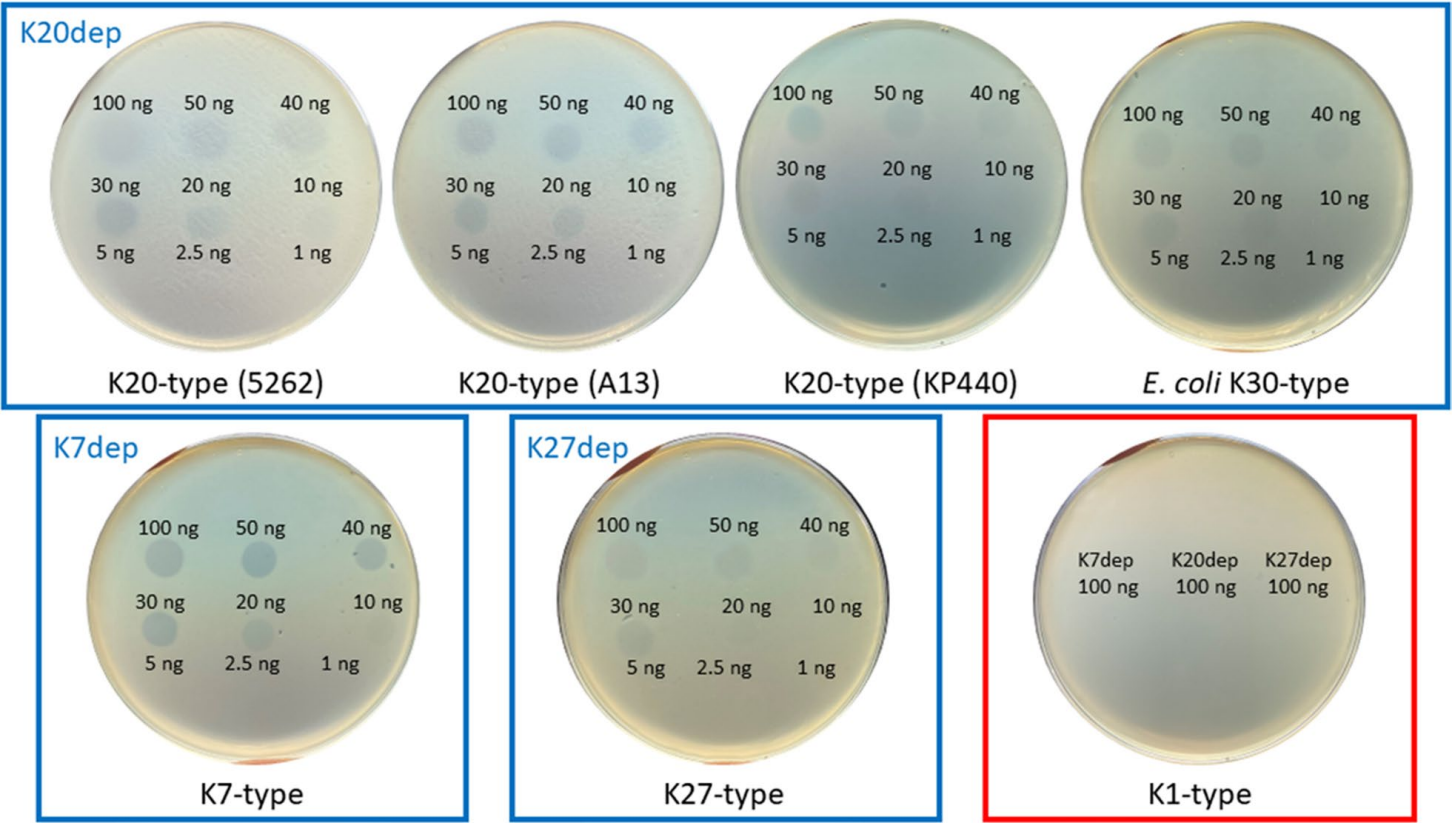
Spot tests of the three capsule depolymerases from phage vB_KpnM‐20, K7dep, K20dep, and K27dep. Four microliters of aliquots containing either 100, 50, 40, 30, 20, 10, 5, 2.5, or 1 ng of K7dep, K20dep, or K27dep capsule depolymerase were spotted on individual lawns of *Klebsiella* K7 (K7ref), K20 (5262, A13, and KP440), and K27-type (K27ref) strains, and an *E. coli* K30-type strain. A K1-type strain (NTUH-K2044) was used as negative control for the three enzymes of which 100 ng were spotted. Semi-clear zones were observed when capsules were digested

The four suspected tail fiber/spike encoded genes, *orf90*, *orf91*, *orf92*, and *orf93*, were expressed in *E. coli.* Construction of expression plasmids containing *orf90*, *orf91*, *orf92*, and *orf93* and expression and purification of the recombinant proteins (SDS-PAGE of ORF90, ORF91, and ORF93) are detailed in the Methods in Additional file 3 and Additional file 2: Fig. S3. The activities of these recombinant proteins were examined using spot tests in the phage vB_KpnM‐20 hosts (*Klebsiella* K7, K27, and K20-type strains and an *E. coli* K30-type strain) and the strains representing other 79 K-types to detect semi-clear spots, indicating capsule digestion. ORF90 (K7dep) and ORF91 (K27dep) generated semi-clear spots on lawns of *Klebsiella* K7-type and K27-type strains, respectively, and ORF93 (K20dep) formed semi-clear spots on the K20-type strains (5262, A13, and KP440) and an *E. coli* K30-type strain. K7dep, K20dep, and K27dep only showed activities against the respective unique *Klebsiella* K-type, and not the other tested K-types (e.g., no spots of 100 ng of K7dep, K20dep, and K27dep were observed on the *Klebsiella* K1-type strain), confirming their specificity. Furthermore, different concentrations of enzymes (100, 50, 40, 30, 20, 10, 5, 2.5, and 1 ng/4 μl) were tested. The results revealed that the minimum concentration of the three enzymes that showed degradation activity was ~ 20 ng in 4 μl SM buffer. No obvious difference was observed among the three K20-type strains and *E. coli* K30-type strain (Fig. 5). In contrast, no enzyme activity was observed for ORF92 on any of the 82 tested *Klebsiella* K-types, and thus ORF92 was named hypothetical protein-1.

### Alcian blue staining of enzyme-treated EPS

**Fig. 6:**
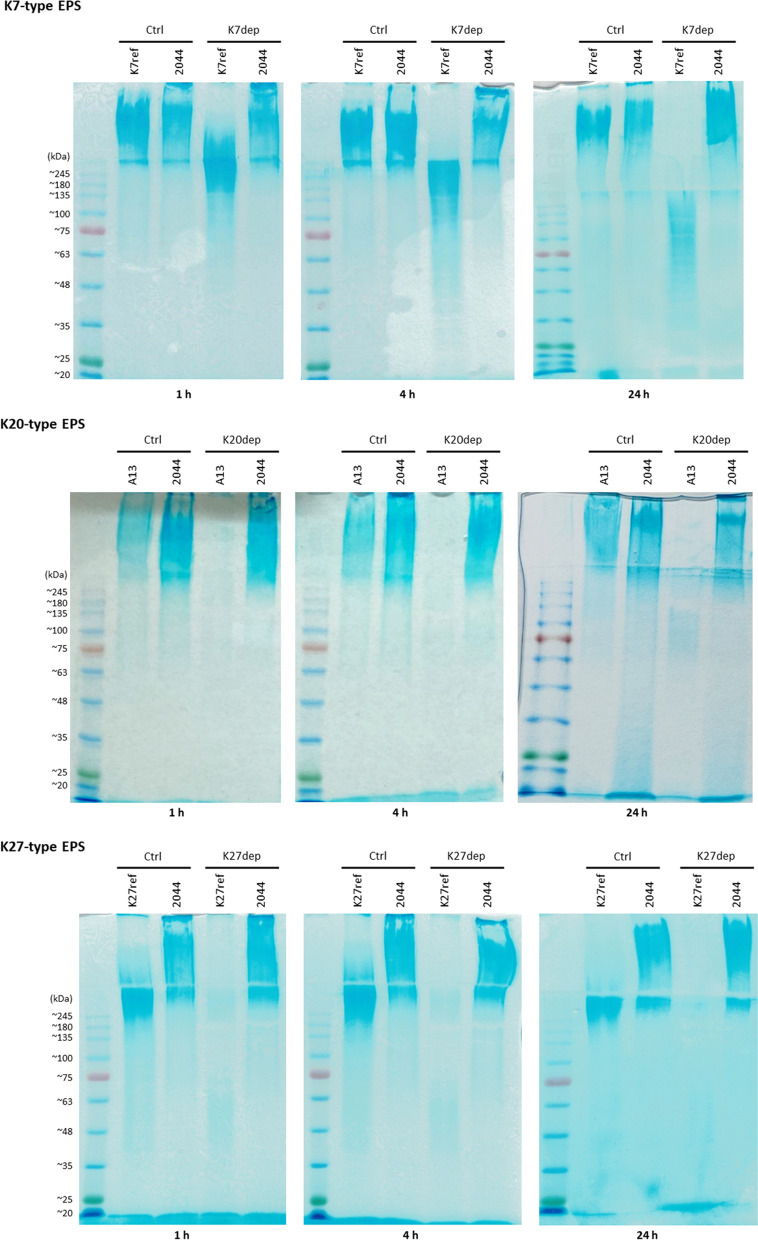
Alcian blue staining of exopolysaccharide treated with K7dep, K20dep, or K27dep. Exopolysaccharide (EPS; 8 μg) extracted from *Klebsiella* K7 (K7ref), K20 (A13), K27 (K27ref), and K1-type (NTUH-K2044) strains was treated with or without 3 μg of K7dep, K20dep, or K27dep in 15 μl of reaction volume at 37 °C for 1, 4, or 24 h and then visualized by Alcian blue stained SDS-PAGE gels. Ctrl (control) indicates no enzyme added. A different capsular-type strain, NTUH-K2044 (K1-type), abbreviated as 2044, was used as a control. BLUeye prestained protein ladder (GeneDireX) was used to indicate molecular weights from ~ 11 to ~ 245 kDa on the gels

The capsule depolymerization activities of K7dep, K20dep, and K27dep were validated through Alcian blue staining of treated EPS (Fig. 6 and Additional file 2: Fig. S4). The results showed that K7, K20, and K27-type EPS was digested by K7dep, K20dep, and K27dep, respectively. The specificity of these enzymes was demonstrated by the insusceptibility of EPS from an irrelevant K-type strain, NTUH-K2044 (K1-type). A time-course effect was observed in K7dep depolymerizing the EPS of K7-type strain for 1, 4, and 24 h, while in K20dep and K27dep, 1 h reaction of the enzymes to the EPS of K20 (A13) and K27-type strain, respectively, showed similar depolymerization activities to the 24 h reaction.

### Therapeutic effect of K20dep in a mouse model

**Fig. 7:**
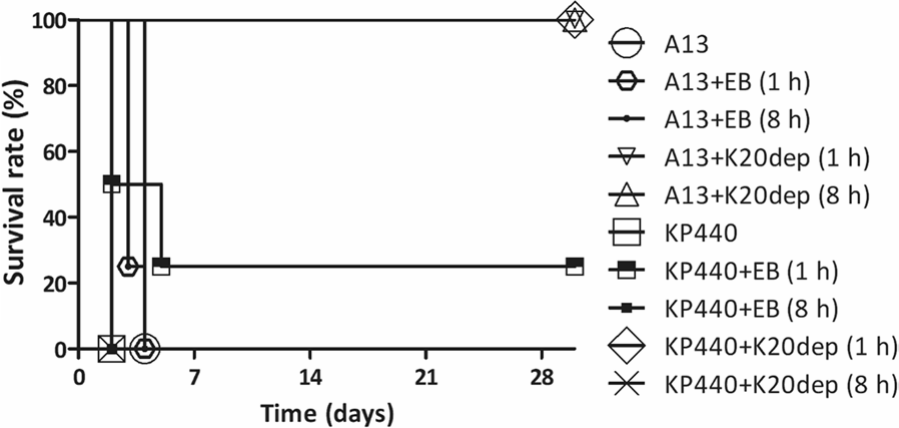
Kaplan–Meier survival curves of A13- and KP440-infected mice with and without K20dep treatment. Four groups of *K. pneumoniae* K20-type-infected mice (A13 + K20dep and KP440 + K20dep, 1 h/8 h) were treated with K20dep, and six other groups (A13 and KP440; A13 + EB and KP440 + EB, 1 h/8 h) were not treated with K20dep. Thirty-day Kaplan–Meier survival curves were generated using GraphPad Prism 5.01

The therapeutic effect of K20dep was evaluated by peritoneal infection of mice with either the K20-type invasive strain A13 (~ 3 × 10^3^ CFU) or the K20-type CRKP strain KP440 (~ 3 × 10^7^ CFU). The effects were assessed at 1 or 8 h post-infection. The results showed that in 1 h post-infection experiments, compared to the control group, enzyme treatment can rescue all mice infected with either A13 or KP440 (mice survived to day 30, without clinical signs of illness; significantly different, p = 0.0082 in both strains), but EB only had no therapeutic effect (even though one mouse infected with KP440 survived after EB treatment, no significant difference was observed between the control group and the KP440 + EB group, p = 0.1266). For 8 h post-infection treatment, similarly, EB only showed no therapeutic effects, whereas enzyme treatment rescued A13-infected mice (p = 0.0082) but not KP440-infected mice (p = 1.0000; Fig. 7).

## Discussion

We isolated a *Klebsiella* bacteriophage, vB_KpnM‐20, and identified three bacteriophage-encoded proteins with capsule depolymerization activities against *K. pneumoniae* K7, K20, and K27-type strains. Interestingly, vB_KpnM‐20 also infected an *E. coli* K30-type strain, although at much lower infectivity. This cross-genus infection is due to the recognition of the surface receptor, capsule, which is almost identical in *Klebsiella* K20-type and *E. coli* K30-type [51]. Accordingly, K20dep also degraded the *E. coli* K30-type capsule. However, surface recognition is not the only determinant of successful lytic infection; nucleic acid injection into the host cell, viral genome and protein synthesis, viral particle assembly, and virion release are also critical. Thus, the differences in phage infectivity toward different strains could be observed.

Comparison of phage genomes with high sequence similarity to phage vB_KpnM‐20 revealed one variable region (n.t.108307–120568), flanked by conserved tail fiber and *vrlC* genes, which contains multiple tail fiber/spike-related genes. We expressed four genes from the variable region of vB_KpnM‐20, *orf90*, *orf91*, *orf92*, and *orf93*, which possibly encode putative tail fibers/spikes and may exhibit capsule depolymerase activities. Even though ORF92 (hypothetical protein-1) showed no activities against the *K. pneumoniae* strains we tested, we cannot exclude the possibility that this protein may have enzymatic activity against an unknown K-type. Our results indicate that genes encoding capsule depolymerases are carried in this variable region in different phages. Thus, PCR amplification of this region from other phages of this group may identify more capsule depolymerases.

The protein expression and purification experiments revealed certain unexpected results. SDS-PAGE showed that the molecular weight of K27dep with the TF tag on pCold plasmid was lower (~ 170 kDa) than its expected weight (194 kDa). Additionally, few proteins with unexpected molecular weights were present; for example, proteins weighing ~ 25 kDa were detected when K7dep (with expected molecular weight of 127 kDa) was purified. Previous studies have proposed that autoproteolytic removal of the C-terminal chaperone domain is necessary for proper folding and kinetically stable conformation of phage proteins, including tail fibers or capsule depolymerases [53, 54]. Thus, we reasoned that shorter-length proteins could result from self-cleavage; however, whether they were part of the targeted recombinant or non-specific binding proteins remains to be determined. No enzyme activity against the specific K-type was observed when the same expression system (the same plasmid and *E. coli* host) was used for the expression of other capsule depolymerases [55]; therefore, it was hypothesized that the enzyme activities were contributed by the protein encoded in the gene that was cloned into the plasmids (and then expressed) rather than the irrelevant proteins co-expressed in the expression experiments. Nevertheless, there is a slight possibility that other proteins with specific capsule depolymerization activities from the *E. coli* expression system interacted with the target protein and were co-purified.

The high specificity of phage vB_KpnM‐20-borne capsule depolymerases, K7dep, K20dep, and K27dep, might be useful for typing *Klebsiella* K7, K20, and K27-type strains, respectively. K20dep can also be used to type *E. coli* K30-type strains. Phage vB_KpnM‐20 targets all the four K-types but not a specific one, and in this case, the phage itself is less appropriate for typing a specific K-type. However, the limitation of either phage or enzyme typing is that bacteria with acapsular mutations resulting in phage-resistance or no recognition by capsule depolymerases would fail to be typed.

Studies of phages that infect *K. pneumoniae* and their potential therapeutic effects have been reported. Lytic *Klebsiella* phages SS [56], NTUH-K2044-K1-1 [24], VTCCBPA43 [57], and vB_KpnM_P-KP2 [58] were shown to effectively treat *K. pneumoniae*-infected animal models. Although phages can continuously propagate and infect their bacterial hosts in vivo, the quality of phages as biological agents is not easy to control, and safety concerns associated with the application of phages in therapeutic use need to be addressed. In addition to direct phage application, phage-encoded enzymes like capsule depolymerases, which can sensitize the targeted K-types to immune or antibiotic attack by breaking down the capsule, have been suggested as another potential therapeutic agent. Compared to phages, phage-borne enzymes are easier to produce and purify. Besides, the depolymerases appeared to be more specific in their actions than phages to target bacteria, which may be beneficial, or at least harmless. To date, capsule depolymerases targeting K1, K2, K3, K5, K8, K11, K13, K19, K21, K22, K23, K25, K30, K35, K37, K47, K51, K56, K57, K63, K64, K69, KN1, KN2, KN3, KN4, and KN5-type *K. pneumoniae* strains have been identified (Additional file 1: Table S4). Some of these have therapeutic effects in vitro and in vivo. Capsule depolymerase Dep42 from *Klebsiella* phage SH-KP152226 showed synergistic effects with polymyxin for degrading K47-type biofilms [59]; K64-ORF41 produced by *Klebsiella* phage SH-KP152410 enhanced the serum and neutrophil killing effects on K64-type strain [60], and both KP32gp37 and KP32gp38 derived from *Klebsiella* phage KP32 sensitized *Klebsiella* K3 and K21-type, respectively, to complement-mediated killing and macrophage phagocytosis [53]. KP32gp37 and KP32gp38 and another capsule depolymerase (depoKP36) from phage KP36, which infects *K. pneumoniae* K63-type, improved the survival of *G. mellonella* larvae infected with enzyme-targeted K-types [53, 61]. Capsule depolymerase K1-ORF34 from the K1-type-targeting phage NTUH-K2044-K1-1, showed therapeutic effects in *K. pneumoniae*-infected mice [24], as did *Klebsiella* phage KpV79-encoded Dep_kpv79 and *Klebsiella* phage KpV767-encoded Dep_kpv767, both targeting K57-type [39]. Dp42 produced by phage vB_KpnP_IME321 was efficient as both a pre- and post-treatment for *K. pneumoniae* KN1-type-challenged/infected mice [22]. Considering these findings and the lack of phage/phage-derived capsule depolymerase targeting *K. pneumoniae* K20-type strains, we thought an investigation of a K20-type-targeting phage and K20-type-specific depolymerase was worthwhile.

An in vivo assay is necessary to test the efficacy of the studied treating agent in the organism. For *K. pneumoniae* infection, *G. mellonella* and mouse are widely applied models; some studies used either of them, and some used both. Compare the advantages of these models, the *G. mellonella* model with cheaper price and less problems related to the ethics is more useful for high-throughput experiments of selection, and the mouse model belonging to mammals as human with more complicated physiological systems is appropriate to assess details in treating effects and outcomes [62–64]. In the current study, a mouse model that had been established for *K. pneumoniae* infection in our lab was applied to evaluate the therapeutic effects of the phage-borne capsule depolymerase K20dep [24].

Our data revealed that early treatment (1 h after infection) for both hypervirulent and carbapenem-resistant K20-type strain infections can effectively save the lives of mice, while late treatment (8 h after infection) only rescued the mice infected with the hypervirulent strain when there was a lower load of initial bacterial inoculation. The load of bacteria in mice and the amount of injected enzyme may alter the enzyme’s ability to treat the infection in a later stage. In this study, we showed that the capsule depolymerase K20dep increased the survival rate of mice infected with K20-type strains, highlighting the potential of K20dep and phage vB_KpnM‐20 for therapeutic development.

There are some limitations to the in vivo assay used in this study. One is that bacteria and the capsule depolymerase were injected at the same site (intra-peritoneum). Whether the treatment effects of K20dep can also be observed when administered through different routes remains unknown. The other limitation is that the treatment was only effective a short time after infection and is better to be validated for longer infection times during which the bacteria would have more time to cause a systemic infection. Volozhantsev et al. demonstrated the differences in mouse survival under treatment with phage-derived capsule depolymerase Dep_kpv74 after 30 min, 3 h, and 24 h of infection (60% of mice were rescued after 3 h and 24 h compared to 90% after 30 min) [38]. Pan et al. administered capsule depolymerase K64dep 1, 8, and 24 h post-infection, with mice treated after 1 h of infection all surviving, while mice treated after 8 h of infection were almost dead [55]. Both studies revealed the reduction in the treatment efficacy of phage-encoded capsule depolymerases when the treatment time is delayed. In the present study, we evaluated the treatment effects of K20dep after 1 and 8 h infection with different strains, and mice infected with strain A13 (hypervirulent K20-type strain) were all alive following treatment. Whether a 24 h-post-infection administration of K20dep would rescue the mice infected with A13 remains unknown.

The clinical significance of K20 as a hypervirulent K-type has been frequently emphasized [30, 34, 65, 66], and drug resistance in K20-type strains poses a serious threat [35, 36, 67–69]. The need for new strategies to combat K20-type is unignorable. *K. pneumoniae* K7 and K27-types have been rarely reported as an infection threat, except that K27-type was once prevalent among multi-drug resistant *K. pneumoniae* in Brazil and among CRKP in Tunisia [70, 71]. In the present study, we identified a phage infecting *K. pneumoniae* K7, K20, and K27-type strains that encodes three capsule depolymerases specific to K7, K20, or K27-type capsules. The phage and its capsule depolymerases exhibited lytic and capsule-degrading effects, and one of the depolymerases reversed the fatal effect of K20-type *K. pneumoniae* infection in mice, providing a foundation for further investigation into possible clinical applications.

## Conclusions

To our knowledge, this is the first study of a phage harboring K7, K20, and K27-type capsule depolymerases. The therapeutic effects of K20dep in an in vivo infection model suggest that phage vB_KpnM‐20 or its depolymerases could be applied as an alternative, non-antibiotic treatment for infections. Considering issues related to safety and specificity, depolymerases are preferable to the phage itself for treatment. The high specificity of the phage-encoded capsule depolymerases, K7dep, K20dep, and K27dep, also suggests their potential for capsular typing, which is important for diagnosis, surveillance, and laboratory studies.

## Supplementary Information

**Additional file 1: Table S1.** *Klebsiella* strains used in this study**. Table S2.** Primers used in this study**. Table S3.** NCBI protein BLAST and HHpred analysis of ORF90, ORF91, ORF92, and ORF93**. Table S4.** *Klebsiella* phage-borne capsule depolymerases.

**Additional file 2: Fig. S1.** Spot tests and efficiency of plating results of phage vB_KpnM‐20 on the plates. **Fig. S2.** Bioinformatic analysis using NCBI Conserved Domain Database and GenomeNet MOTIF Search against Pfam library. **Fig. S3.** Coomassie blue staining of SDS-PAGE gels showing the process of protein expression and purification of K7dep (orf90), K20dep (orf93), and K27dep (orf91). **Fig. S4.** Original images of SDS-PAGE gels stained with Alcian blue of purified EPS treated with K7dep, K20dep, and K27dep for 1, 4, or 24 h.

**Additional file 3.** **Methods.** Plasmid construction**.** Expression and purification of putative capsule depolymerases. Coomassie blue staining and quantification of capsule depolymerases. **Results.** NCBI protein BLAST-Multiple Sequence Alignment viewer

## Data Availability

All data generated or analyzed during this study are included in this published article and its supplementary information files.

## Abbreviations

CRKP: Carbapenem-resistant *K. pneumoniae*
K-types: Capsular types
LB: Luria–Bertani
EOP: Efficiency of plating
CFU: Colony-forming units
EPS: Exopolysaccharide
LD_50_: Median lethal dose
EB: Elution buffer
