# Supplementary material for: Identification of three capsule depolymerases in a bacteriophage infecting Klebsiella pneumoniae capsular types K7, K20, and K27 and therapeutic application
Source: J Biomed Sci. 2023 May 20;30:31. doi: 10.1186/s12929-023-00928-0 (PMC10199534; doi:10.1186/s12929-023-00928-0)
Supplement: Supplementary file 1 — Additional file 1: Table S1. Klebsiella strains used in this study. Table S2. Primers used in this study. Table S3. NCBI protein BLAST and HHpred analysis of ORF90, ORF91, ORF92, and ORF93. Table S4. Klebsiella phage-borne capsule depolymerases. [file 12929_2023_928_MOESM1_ESM.docx]

**Table S1. *Klebsiella* strains used in this study**

| Capsular type | Strain | Species | Note |
| --- | --- | --- | --- |
| K1 | A5054 | *K. pneumoniae* | Reference strain |
|  | NTUH-K2044 | *K. pneumoniae* | [1] |
| K2 | B5055 | *K. pneumoniae* | Reference strain |
| K3 | C5046 | *K. pneumoniae* | Reference strain |
| K4 | D5050 | *K. pneumoniae* subsp. *ozaenae* | Reference strain |
| K5 | E5051 | *K. pneumoniae* subsp. *ozaenae* | Reference strain |
| K6 | F052 | *K. pneumoniae* subsp. *ozaenae* | Reference strain |
| K7 | Aerogenes 4140 | *K. pneumoniae* | Reference strain |
| K8 | *Klebsiella* 1015 | *K. pneumoniae* | Reference strain |
| K9 | *Klebsiella* 1056 | *K. pneumoniae* | Reference strain |
| K10 | *Klebsiella* 919 | *K. pneumoniae* | Reference strain |
| K11 | *Klebsiella* 390 | *K. pneumoniae* | Reference strain |
| K12 | *Klebsiella* 313 | *K. pneumoniae* | Reference strain |
| K13 | *Klebsiella* 1470 | *K. pneumoniae* | Reference strain |
| K14 | 138 | *K.* (*Raoultella*) *planticola* | Reference strain |
| K15 | Mich. 61 | *K. pneumoniae* | Reference strain |
| K16 | 2069/49 | *K. pneumoniae* | Reference strain |
| K17 | 2005/49 | *K. pneumoniae* | Reference strain |
| K18 | 1754/49 | *K. pneumoniae* | Reference strain |
| K19 | 293/50 | *K. pneumoniae* | Reference strain |
| K20 | A13 (NTUH-KP13) | *K. pneumoniae* | [2] |
|  | 5262 | *K. pneumoniae* | [2] |
|  | KP440 | *K. pneumoniae* | Unpublished strain |
| K21 | 1702/49 | *K. pneumoniae* | Reference strain |
| K22 | 1996/49 | *K. pneumoniae* | Reference strain |
| K23 | 2812/50 | *K. pneumoniae* | Reference strain |
| K24 | 1680/49 | *K. pneumoniae* | Reference strain |
| K25 | 2002/49 | *K. pneumoniae* | Reference strain |
| K26 | 5884 | *K. oxytoca* | Reference strain |
| K27 | 6613 | *K. pneumoniae* | Reference strain |
| K28 | 5758 | *K. pneumoniae* | Reference strain |
| K29 | 5725y | *K. oxytoca* | Reference strain |
| K30 | 7824 | *K. pneumoniae* | Reference strain |
| K31 | 6258 | *K. pneumoniae* | Reference strain |
| K32 | 6837 | *K.* (*Raoultella*) *ornithinolytica* | Reference strain |
| K33 | 6168 | *K. pneumoniae* | Reference strain |
| K34 | 7522 | *K. pneumoniae* | Reference strain |
| K35 | 7444 | *K.* (*Raoultella*) *planticola* | Reference strain |
| K36 | 8306 | *K. pneumoniae* | Reference strain |
| K37 | 8238 | *K. pneumoniae* | Reference strain |
| K38 | 8414 | *K. pneumoniae* | Reference strain |
| K39 | 7749 | *K. pneumoniae* | Reference strain |
| K40 | 8588 | *K. pneumoniae* | Reference strain |
| K41 | 6177 | *K. michiganensis* | Reference strain |
| K42 | 1702 | *K. pneumoniae* | Reference strain |
| K43 | 2482 | *K. pneumoniae* | Reference strain |
| K44 | 7730 | *K.* (*Raoultella*) *ornithinolytica* | Reference strain |
| K45 | 8464 | *K. pneumoniae* | Reference strain |
| K46 | 5281 | *K. pneumoniae* | Reference strain |
| K47 | 9682 | *K. pneumoniae* | Reference strain |
| K48 | 1196 | *K. variicola* | Reference strain |
| K49 | 6115 | *K. variicola* | Reference strain |
| K50 | 1303/50 | *K. pneumoniae* II-B | Reference strain |
| K51 | 4715/50 | *K. pneumoniae* | Reference strain |
| K52 | 5759/50 | *K. pneumoniae* | Reference strain |
| K53 | 1756/51 | *K. variicola* | Reference strain |
| K54 | Stanley | *K. variicola* | Reference strain |
| K55 | 3985/51 | *K. pneumoniae* | Reference strain |
| K56 | 3534/51 | *K. variicola* | Reference strain |
| K57 | 4425/51 | *K. variicola* | Reference strain |
| K58 | 636/52 | *K. variicola* | Reference strain |
| K59 | 2212/52 | *K. michiganensis* | Reference strain |
| K60 | 4463/52 | *K. pneumoniae* II-B | Reference strain |
| K61 | 5710/52 | *K. pneumoniae* | Reference strain |
| K62 | 5711/52 | *K. pneumoniae* | Reference strain |
| K63 | 5845/52 | *K. pneumoniae* | Reference strain |
| K64 | NCTC 8172 | *K. pneumoniae* | Reference strain |
| K65 | SW4 | *K.* (*Raoultella*) *terrigena* | Reference strain |
| K66 | 438(3a) | *K. michiganensis* | Reference strain |
| K67 | 264(1) | *K.* (*Raoultella*) *terrigena* | Reference strain |
| K68 | 265(1) | *K.* (*Raoultella*) *terrigena* | Reference strain |
| K69 | 889 | *K.* (*Raoultella*) *terrigena* | Reference strain |
| K70 | 167 | *K. michiganensis* | Reference strain |
| K71 | 4349 | *K. variicola* | Reference strain |
| K72 | 1205 | *K.* (*Raoultella*) *ornithinolytica* | Reference strain |
| K74 | 371 | *K. oxytoca* | Reference strain |
| K79 | 325 | *K.* (*Raoultella*) *planticola* | Reference strain |
| K80 | 708 | *K. pneumoniae* II-B | Reference strain |
| K81 | 370 | *K. pneumoniae* | Reference strain |
| K82 | 3454-70 | *K. pneumoniae* | Reference strain |
| KN1 | A1517 | *K. pneumoniae* | [3] |
|  | 6451N | *K. pneumoniae* | [4] |
|  | Ca0514 | *K. pneumoniae* | [4] |
| KN2 | Ca0507 | *K. pneumoniae* | [5] |
| KN3 | N386-KCR59 | *K. pneumoniae* | [6] |
|  | N345-2-KCR57 | *K. pneumoniae* | [6] |
|  | N348-KCR58 | *K. pneumoniae* | [6] |
|  | 1595E | *K. pneumoniae* | [4] |
|  | 2283219 | *K. pneumoniae* | [4] |
| KN4 | 1461 | *K. pneumoniae* | [2] |
|  | 4565 | *K. pneumoniae* | [4] |
|  | 4486-2 | *K. pneumoniae* | [4] |
|  | 7966E | *K. pneumoniae* | [4] |
|  | 2139670 | *K. pneumoniae* | [4] |
|  | 3669933 | *K. pneumoniae* | [4] |
| KN5 | Ca0431 | *K. pneumoniae* | [7] |

**Table S2. Primers used in this study**

| Primer name | Sequences (5' to 3') | Purpose |
| --- | --- | --- |
| k27f | ATCGTTTCTGATGCATGGG | phage DNA termini detection |
| k27r | CAAGTGATAACACGATGCC | phage DNA termini detection |
| K27 S1-1-F | ATGGCTAATAAACCCACGAAAC | K7dep (*orf90*) expression |
| K27 S1-1-R | AGAAAATCGGGCTTCCAAGG | K7dep (*orf90*) expression |
| K27-tail-NdeI-F | GAACCACATATGAAAACGCAATTTAACCAATC | K20dep (*orf93*) expression |
| K27-tail-HindIII-R | TTTAAAAGCTTTTAACCGATTTTGATTGTATATG | K20dep (*orf93*) expression |
| phage K27 S1-2 (Nhe1,Sac1)F1 | TACCCCATATGCTAG CGAGCTCATGGGGTATTTTCAAATGAC | K27dep (*orf91*) expression |
| phage K27 S1-2 (Xho1)R1 | GGGGGCGTCTCGAGTTATTTAAATTTACTTTCTAAATC | K27dep (*orf91*) expression |
| K27-S1-3-Nde1-F | CAGGAGATTCCATATGAATCCACAATTCAGTCAGC | HP1 (*orf92*) expression |
| K27-S1-3-Xho1-R(stop) | GGCTTGCGACAATCTCGAGCTACTGTGGTAGTAGAGTTG | HP1 (*orf92*) expression |

**Table S3. NCBI protein BLAST and HHpred analysis of ORF90, ORF91, ORF92, and ORF93**

| Protein | Matched proteins (NCBI protein BLAST) | Accession no. | Coverage; Identity |
| --- | --- | --- | --- |
| ORF90 | tail protein^a^ [*Klebsiella* phage May] | YP_009796159.1 | 100%; 99.58% |
| ORF91 | tail fiber protein^a^ [*Klebsiella* phage May] | YP_009796160.1 | 99%; 97.75% |
| ORF92 | hypothetical protein^a^ [*Klebsiella* phage K751] | UPW36206.1 | 83%; 88.91% |
|  | phage tail fiber protein [*K. pneumoniae*] | WP_181502371.1 | 86%; 72.83% |
| ORF93 | hypothetical protein^a^ [*Klebsiella* phage K751] | UPW36008.1 | 100%; 99.31% |
|  | tail spike protein [*Klebsiella* phage vB_KpnM_KpS110] | YP_009798897.1 | 11%; 84.88% (Query residue 1-86) |
|  | non-contractile tail fiber protein [*Klebsiella* phage vB_KpnS-VAC70] | UEW68236.1 | 84%; 67.59% (Query residue 114-722) |

^a^Best matched protein sequences

| Protein | Alignment^a^ (HHpred) | Query Position | Probability; E-value |
| --- | --- | --- | --- |
| ORF90 | tail spike protein [*Escherichia* virus CBA120] | residue 1-338 | 100%; 1.9e-34 |
|  | tail spike protein [*Acinetobacter* phage vB_AbaP_AS12] | residue 436-1176 | 99.87%; 2.2e-18 |
| ORF91 | tail spike protein [*Acinetobacter* phage vB_AbaP_AS12] | residue 1093-1290 | 99.85%; 3.6e-17 |
|  | exopolysaccharide biosynthesis protein (tail spike-like) [*Pantoea stewartii* subsp. *stewartii* DC283] | residue 14-641 | 99.57%; 8.9e-13 |
| ORF92 | tail spike protein [*Escherichia* phage Cba120] | residue 133-599 | 99.9%; 1.3e-19 |
| ORF93 | tail spike protein [*Escherichia* virus CBA120] | residue 146-348 | 98.93%; 1.7e-7 |

^a^Protein sequences with the highest probability in certain regions

**Table S4. *Klebsiella* phage-borne capsule depolymerases**

| *Klebsiella* phage | Depolymerase | Accession No. | K-type targeted | Reference |
| --- | --- | --- | --- | --- |
| 0507-KN2-1 | ORF96 | YP_008532047.1 | KN2 | [5] |
| NTUH-K2044-K1-1 | K1-ORF34 | YP_009098385.1 | K1 | [1] |
| KP36 | depoKP36 | YP_009226011.1 | K63 | [8] |
| K5-2 | K5-2 ORF37 | APZ82804.1 | K30/K69 | [9] |
|  | K5-2 ORF38 | APZ82805.1 | K5 | [9] |
| K5-4 | K5-4 ORF37 | APZ82847.1 | K8 | [9] |
|  | K5-4 ORF38 | APZ82848.1 | K5 | [9] |
| K64-1 | S1-1 | YP_009153197.1 | K11 | [7] |
|  | S1-2 | YP_009153195.1 | KN4 | [7] |
|  | S1-3 | YP_009153196.1 | K21 | [7] |
|  | S2-1 | YP_009153198.1 | KN5 | [7] |
|  | S2-2 | YP_009153199.1 | K25 | [7] |
|  | S2-3 | YP_009153200.1 | K35 | [7] |
|  | S2-4 | YP_009153201.1 | K1 | [7] |
|  | S2-5 | YP_009153202.1 | K64 | [7] |
|  | S2-6 | YP_009153203.1 | K30/K69 | [7] |
| KP32 | KP32gp37 | YP_003347555.1 | K3 | [10] |
|  | KP32gp38 | YP_003347556.1 | K21 | [10] |
| KpV41 | kpv41_46 | ALO80736 | K1 | [11] |
|  | kpv41_55 | KT964103 | N.A. | [11] |
| KpV71 | Dep_kpv71 (kpv71_52) | YP_009302756.1 | K1 | [11] |
| KpV74 | Dep_kpv74 (kpv74_56) | APZ82768.1 | K2/K13 | [11, 12] |
| KN1-1 | KN1dep | BBF66844.1 | KN1 | [4] |
| KN3-1 | KN3dep | BBF66867.1 | KN3 | [4] |
|  | K56dep | BBF66868.1 | K56 | [4] |
| KN4-1 | KN4dep | BBF66888.1 | KN4 | [4] |
| vB_KpnP_IME321 | Dp42 | AXE28435 | KN1 | [13] |
| SH-KP152226 | Dep42 | QDF14644.1 | K47 | [14] |
| πVLC5 | ORF49 | QIW86419.1 | K22/K37 | [15] |
|  | ORF58 | QIW86428.1 | N.A. | [15] |
| πVLC6 | ORF51 | QJI52623.1 | K22/K37 | [15] |
|  | ORF58 | QJI52632.1 | K13 | [15] |
| IME205 | Dpo42 | YP_009785899.1 | K47 | [16] |
|  | Dpo43 | YP_009785900.1 | K47 | [16] |
| KpV79 | Dep_kpv79 | ATI16495.1 | K57 | [17] |
| KpV767 | Dep_kpv767 | AOZ65519.1 | K57 | [17] |
| SH-KP152410 | K64-ORF41 | YP_009797016.1 | K64 | [18] |
| B1 | B1dep (ORF61) | QTP95996.1 | K2/K13 | [19] |
| GBH001 | GBH001_056 | N.A. | K1 | [20] |
| GBH038 | GBH038_054 | N.A. | K2 | [20] |
| GBH019 | GBH019_279 | N.A. | K51 | [20] |
| vB_KpP_TUN1 | gp47 | CAF0683093.1 | K64 | [21] |
| vB_KpnP_Dlv622 | Dep622 | QOI68577.1 | K23 | [22] |
| KpS8 | DepS8 | QIW88225.1 | K23 | [22] |
| RAD2 | DpK2 | QUU29414.1 | K2 | [23] |
| P510 | P510dep | QOV05454.1 | K64 | [24] |
| 1611E-K2-1 | K2-ORF16 | ATS92567.1 | K2 | [25] |
| P560 | P560dep (ORF43) | QOV05502.1 | K47 | [26] |
| SH-KP156570 | K19-Dpo41 | N.A. | K19 | [27] |
| KP34 | KP34p57 | YP_003347643.1 | K63 | [28] |
| vB_KpnP_ZX1 | Dep_ZX1 (ORF60) | QTH79846.1 | K57 | [29] |

N.A., not available.

**References**

1. Lin TL, Hsieh PF, Huang YT, Lee WC, Tsai YT, Su PA, et al. Isolation of a bacteriophage and its depolymerase specific for K1 capsule of *Klebsiella pneumoniae*: implication in typing and treatment. J Infect Dis. 2014;210(11):1734-44.
2. Pan YJ, Lin TL, Chen YH, Hsu CR, Hsieh PF, Wu MC, et al. Capsular types of *Klebsiella pneumoniae* revisited by *wzc* sequencing. PLoS One. 2013;8(12):e80670.
3. Pan YJ, Fang HC, Yang HC, Lin TL, Hsieh PF, Tsai FC, et al. Capsular polysaccharide synthesis regions in *Klebsiella pneumoniae* serotype K57 and a new capsular serotype. J Clin Microbiol. 2008;46(7):2231-40.
4. Pan YJ, Lin TL, Chen YY, Lai PH, Tsai YT, Hsu CR, et al. Identification of three podoviruses infecting *Klebsiella* encoding capsule depolymerases that digest specific capsular types. Microb Biotechnol. 2019;12(3):472-86.
5. Hsu CR, Lin TL, Pan YJ, Hsieh PF, Wang JT. Isolation of a bacteriophage specific for a new capsular type of *Klebsiella pneumoniae* and characterization of its polysaccharide depolymerase. PLoS One. 2013;8(8):e70092.
6. Pan YJ, Lin TL, Lin YT, Su PA, Chen CT, Hsieh PF, et al. Identification of Capsular Types in Carbapenem-Resistant *Klebsiella pneumoniae* Strains by *wzc* Sequencing and Implications for Capsule Depolymerase Treatment. Antimicrob Agents Chemother. 2015;59(2):1038-47.
7. Pan YJ, Lin TL, Chen CC, Tsai YT, Cheng YH, Chen YY, et al. *Klebsiella* Phage ΦK64-1 Encodes Multiple Depolymerases for Multiple Host Capsular Types. J Virol. 2017;91(6):e02457-16.
8. Majkowska-Skrobek G, Łątka A, Berisio R, Maciejewska B, Squeglia F, Romano M, et al. Capsule-Targeting Depolymerase, Derived from *Klebsiella* KP36 Phage, as a Tool for the Development of Anti-Virulent Strategy. Viruses. 2016;8(12):324.
9. Hsieh PF, Lin HH, Lin TL, Chen YY, Wang JT. Two T7-like Bacteriophages, K5-2 and K5-4, Each Encodes Two Capsule Depolymerases: Isolation and Functional Characterization. Sci Rep. 2017;7(1):4624.
10. Majkowska-Skrobek G, Latka A, Berisio R, Squeglia F, Maciejewska B, Briers Y, et al. Phage-Borne Depolymerases Decrease *Klebsiella pneumoniae* Resistance to Innate Defense Mechanisms. Front Microbiol. 2018;9:2517.
11. Solovieva EV, Myakinina VP, Kislichkina AA, Krasilnikova VM, Verevkin VV, Mochalov VV, et al. Comparative genome analysis of novel Podoviruses lytic for hypermucoviscous *Klebsiella pneumoniae* of K1, K2, and K57 capsular types. Virus Res. 2018;243:10-8.
12. Volozhantsev NV, Borzilov AI, Shpirt AM, Krasilnikova VM, Verevkin VV, Denisenko EA, et al. Comparison of the therapeutic potential of bacteriophage KpV74 and phage-derived depolymerase (β-glucosidase) against *Klebsiella pneumoniae* capsular type K2. Virus Res. 2022;322:198951.
13. Wang C, Li P, Niu W, Yuan X, Liu H, Huang Y, et al. Protective and therapeutic application of the depolymerase derived from a novel KN1 genotype of *Klebsiella pneumoniae* bacteriophage in mice. Res Microbiol. 2019;170(3):156-64.
14. Wu Y, Wang R, Xu M, Liu Y, Zhu X, Qiu J, et al. A Novel Polysaccharide Depolymerase Encoded by the Phage SH-KP152226 Confers Specific Activity Against Multidrug-Resistant *Klebsiella pneumoniae* via Biofilm Degradation. Front Microbiol. 2019;10:2768.
15. Domingo-Calap P, Beamud B, Mora-Quilis L, González-Candelas F, Sanjuán R. Isolation and Characterization of Two *Klebsiella pneumoniae* Phages Encoding Divergent Depolymerases. Int J Mol Sci. 2020;21(9):3160.
16. Liu Y, Leung SSY, Huang Y, Guo Y, Jiang N, Li P, et al. Identification of Two Depolymerases From Phage IME205 and Their Antivirulent Functions on K47 Capsule of *Klebsiella pneumoniae*. Front Microbiol. 2020;11:218.
17. Volozhantsev NV, Shpirt AM, Borzilov AI, Komisarova EV, Krasilnikova VM, Shashkov AS, et al. Characterization and Therapeutic Potential of Bacteriophage-Encoded Polysaccharide Depolymerases with β Galactosidase Activity against *Klebsiella pneumoniae* K57 Capsular Type. Antibiotics (Basel). 2020;9(11):732.
18. Li J, Sheng Y, Ma R, Xu M, Liu F, Qin R, et al. Identification of a Depolymerase Specific for K64-Serotype *Klebsiella pneumoniae*: Potential Applications in Capsular Typing and Treatment. Antibiotics (Basel). 2021;10(2):144.
19. Pertics BZ, Cox A, Nyúl A, Szamek N, Kovács T, Schneider G. Isolation and Characterization of a Novel Lytic Bacteriophage against the K2 Capsule-Expressing Hypervirulent *Klebsiella pneumoniae* Strain 52145, and Identification of Its Functional Depolymerase. Microorganisms. 2021;9(3):650.
20. Blundell-Hunter G, Enright MC, Negus D, Dorman MJ, Beecham GE, Pickard DJ, et al. Characterisation of Bacteriophage-Encoded Depolymerases Selective for Key *Klebsiella pneumoniae* Capsular Exopolysaccharides. Front Cell Infect Microbiol. 2021;11:686090.
21. Eckstein S, Stender J, Mzoughi S, Vogele K, Kühn J, Friese D, et al. Isolation and characterization of lytic phage TUN1 specific for *Klebsiella pneumoniae* K64 clinical isolates from Tunisia. BMC Microbiol. 2021;21(1):186.
22. Gorodnichev RB, Volozhantsev NV, Krasilnikova VM, Bodoev IN, Kornienko MA, Kuptsov NS, et al. Novel *Klebsiella pneumoniae* K23-Specific Bacteriophages From Different Families: Similarity of Depolymerases and Their Therapeutic Potential. Front Microbiol. 2021;12:669618.
23. Dunstan RA, Bamert RS, Belousoff MJ, Short FL, Barlow CK, Pickard DJ, et al. Mechanistic Insights into the Capsule-Targeting Depolymerase from a *Klebsiella pneumoniae* Bacteriophage. Microbiol Spectr. 2021;9(1):e0102321.
24. Li M, Li P, Chen L, Guo G, Xiao Y, Chen L, et al. Identification of a phage-derived depolymerase specific for KL64 capsule of *Klebsiella pneumoniae* and its anti-biofilm effect. Virus Genes. 2021;57(5):434-42.
25. Lin TL, Yang FL, Ren CT, Pan YJ, Liao KS, Tu IF, et al. Development of *Klebsiella pneumoniae* Capsule Polysaccharide-Conjugated Vaccine Candidates Using Phage Depolymerases. Front Immunol. 2022;13:843183.
26. Li M, Wang H, Chen L, Guo G, Li P, Ma J, et al. Identification of a phage-derived depolymerase specific for KL47 capsule of *Klebsiella pneumoniae* and its therapeutic potential in mice. Virol Sin. 2022;37(4):538-46.
27. Hua Y, Wu Y, Guo M, Ma R, Li Q, Hu Z, et al. Characterization and Functional Studies of a Novel Depolymerase Against K19-Type *Klebsiella pneumoniae*. Front Microbiol. 2022;13:878800.
28. Smug BJ, Majkowska-Skrobek G, Drulis-Kawa Z. PhREEPred: Phage Resistance Emergence Prediction Web Tool to Foresee Encapsulated Bacterial Escape from Phage Cocktail Treatment. J Mol Biol. 2022;434(14):167670.
29. Li P, Ma W, Shen J, Zhou X. Characterization of Novel Bacteriophage vB_KpnP_ZX1 and Its Depolymerases with Therapeutic Potential for K57 *Klebsiella pneumoniae* Infection. Pharmaceutics. 2022;14(9):1916.
