# Supplementary material for: Identification of three capsule depolymerases in a bacteriophage infecting Klebsiella pneumoniae capsular types K7, K20, and K27 and therapeutic application
Source: J Biomed Sci. 2023 May 20;30:31. doi: 10.1186/s12929-023-00928-0 (PMC10199534; doi:10.1186/s12929-023-00928-0)
Supplement: Supplementary file 2 — Additional file 2: Fig. S1. Spot tests and efficiency of plating results of phage vB_KpnM‐20 on the plates. Fig. S2. Bioinformatic analysis using NCBI Conserved Domain Database and GenomeNet MOTIF Search against Pfam library. Fig. S3. Coomassie blue staining of SDS-PAGE gels showing the process of protein expression and purification of K7dep (orf90), K20dep (orf93), and K27dep (orf91). Fig. S4. Original images of SDS-PAGE gels stained with Alcian blue of purified EPS treated with K7dep, K20dep, and K27dep for 1, 4, or 24 h. [file 12929_2023_928_MOESM2_ESM.docx]

**Additional file 1: Figure legends**

**a**


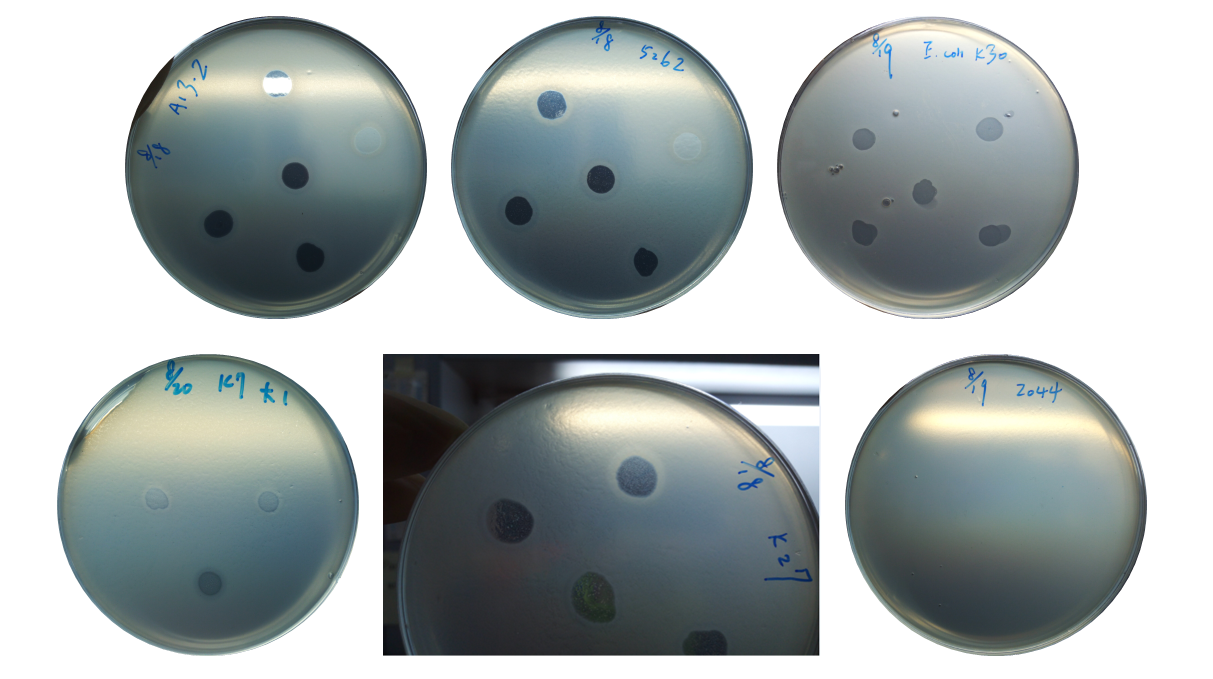


**b**


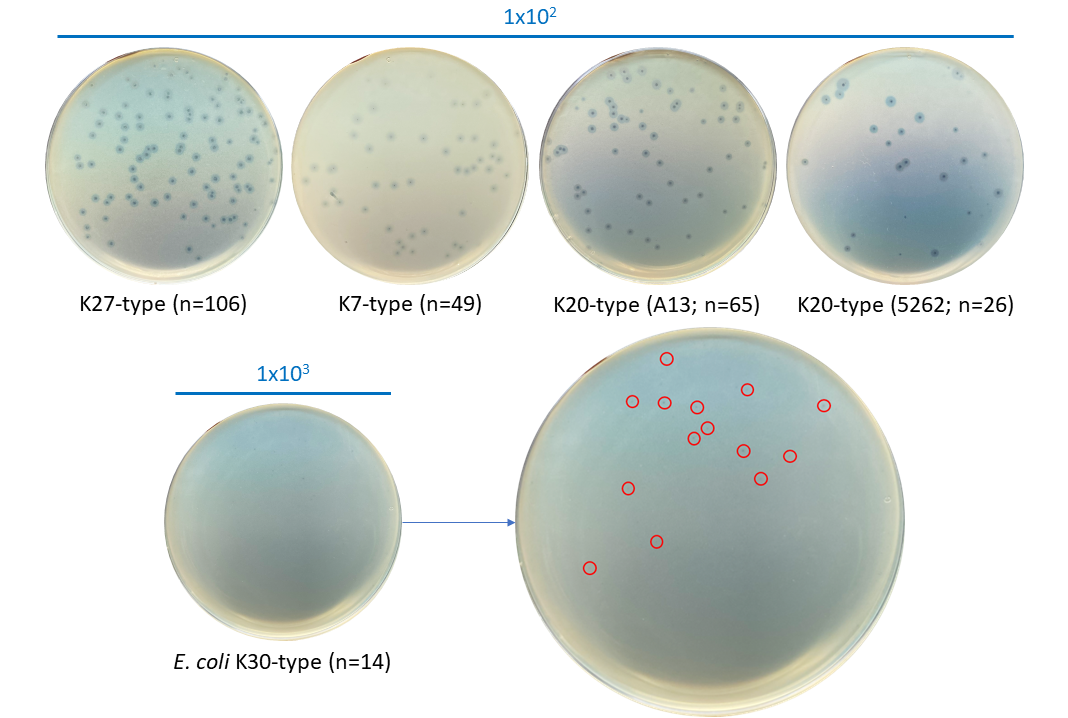


**Fig. S1**

**Fig. S1. Spot tests and efficiency of plating (EOP) results of phage vB_KpnM‐20 on the plates**

**a**. Original (uncropped) plate images of phage vB_KpnM‐20 (10^8^ PFU) spotted on lawns of K7, K20 (A13 and 5262), K27, and K1-type (NTUH-K2044) strains and an *E. coli* K30-type strain for **Fig. 1**; **b**. Difference in infection efficiency of phage vB_KpnM‐20 on *Klebsiella* K27, K7, K20-type (A13 and 5262) strains and an *E. coli* K30-type strain shown on the plates from one of the three independent experiments. Here, we show an example of ~1×10^2^ PFU of phage infecting the K27, K7, and K20-type strains (100 μl from 1×10^3^ PFU/ml of phage suspension was mixed with different bacterial strains and spread on the agar plate) and ~1×10^3^ PFU of phage infecting the *E. coli* K30-type strain (no plaques were seen for ~1×10^2^ PFU experiment). Phage plaques on the lawn of the *E. coli* K30-type strain were too small to see and are marked with red circles. The phage titer was determined using the K27-type strain on which EOP was set to 100%. To calculate the EOP for other strains, for example, the ratio for strain A13 was 61.3% (65/106) compared to the K27-type strain.

**a**


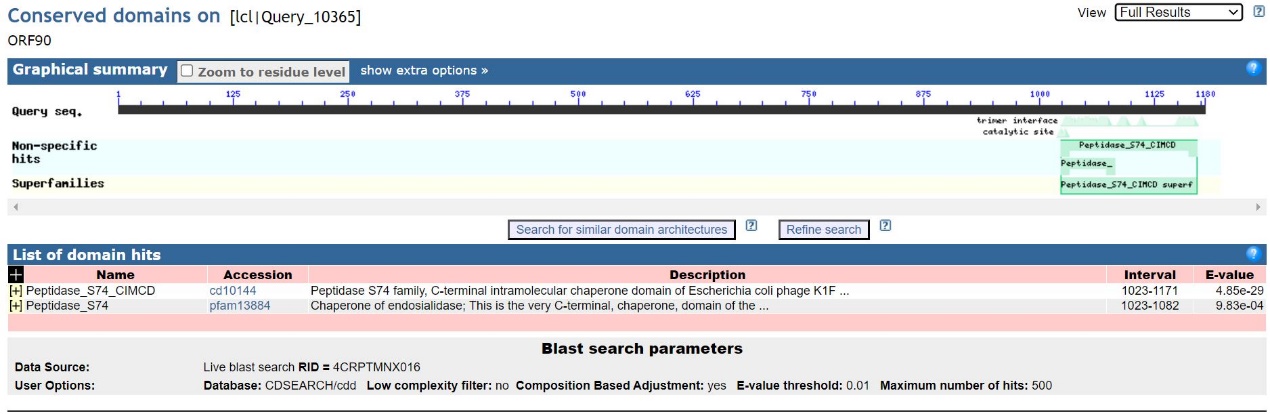


**b**


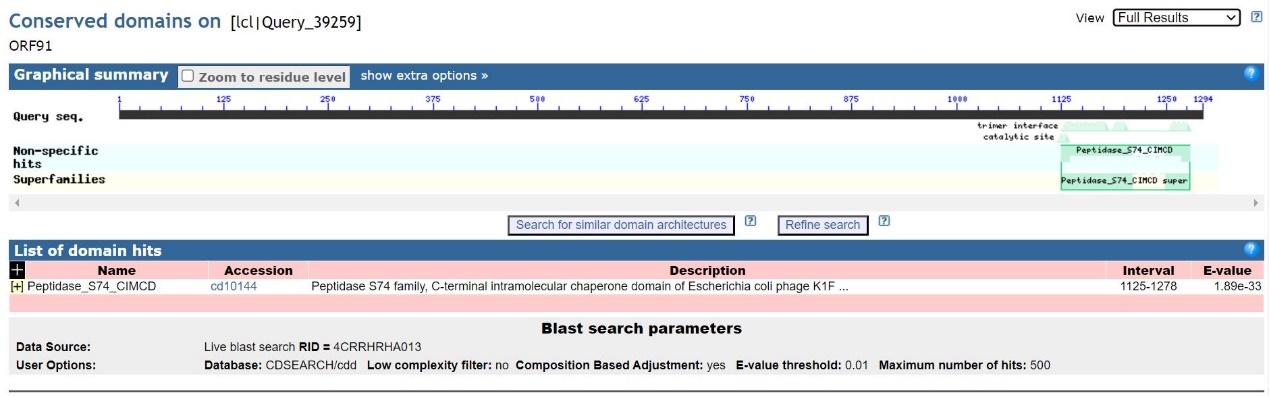


**c**


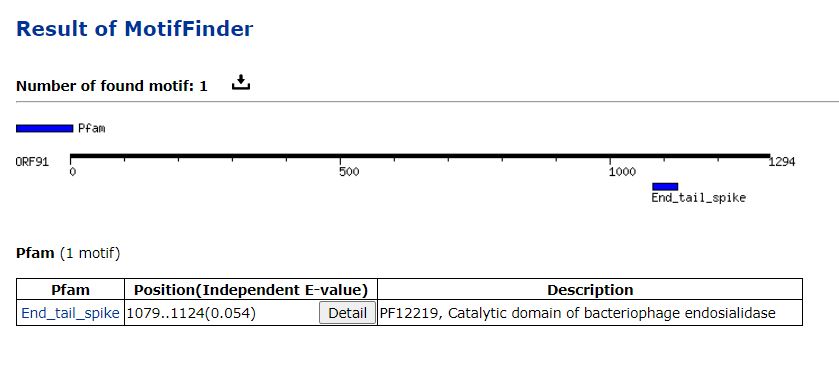


**Fig. S2**

**Fig. S2. Bioinformatic analysis using NCBI Conserved Domain Database (CDD) and GenomeNet MOTIF Search (MotifFinder) against Pfam library**

**a** and **b**. Peptidase of C-terminal intramolecular chaperone domain was identified in ORF90 and ORF91; **c**. One motif of catalytic domain of bacteriophage endosialidase from Pfam was found in ORF91.


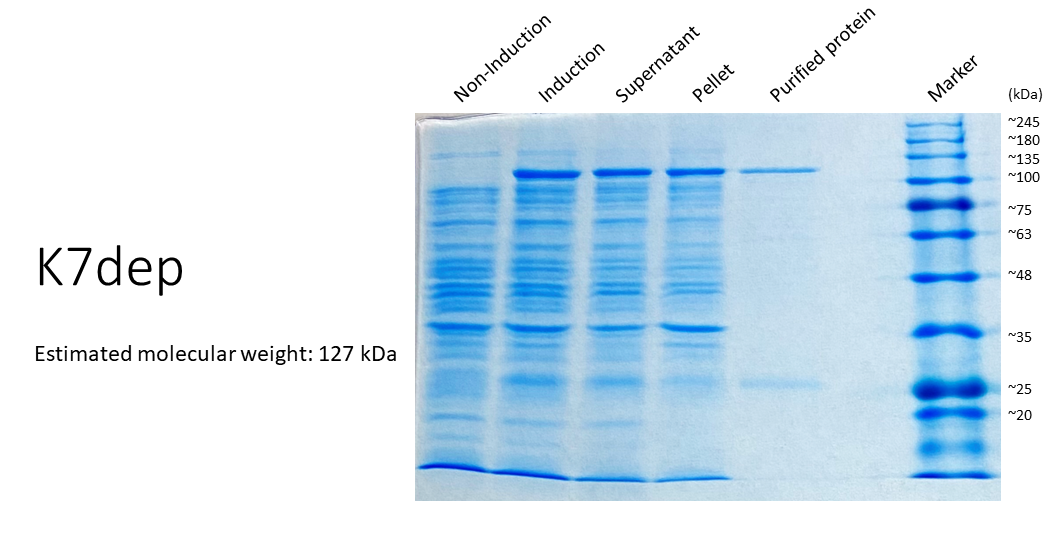


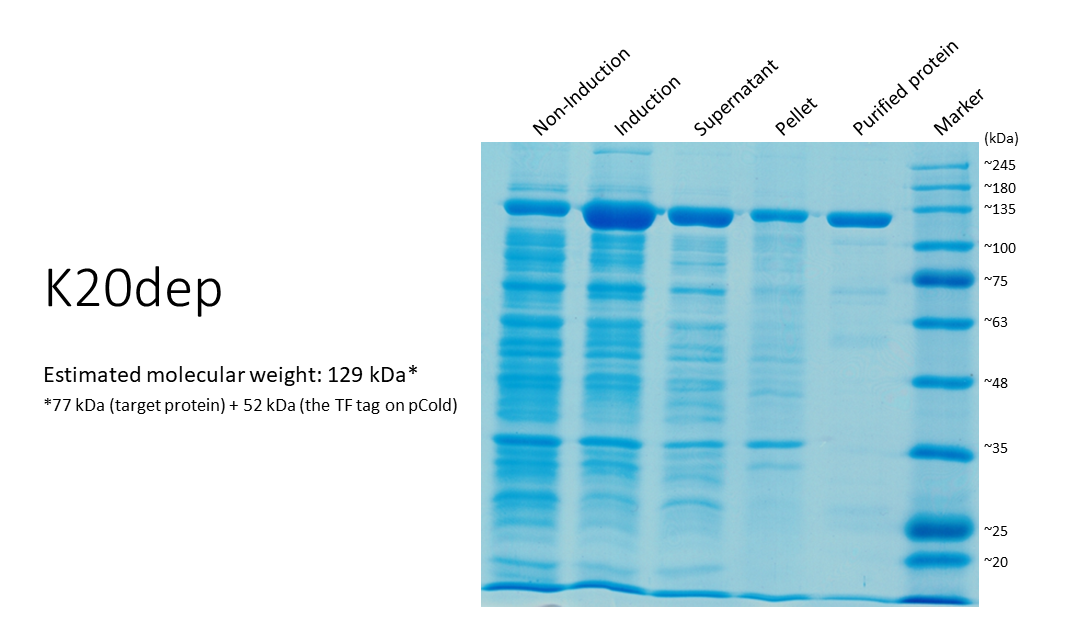


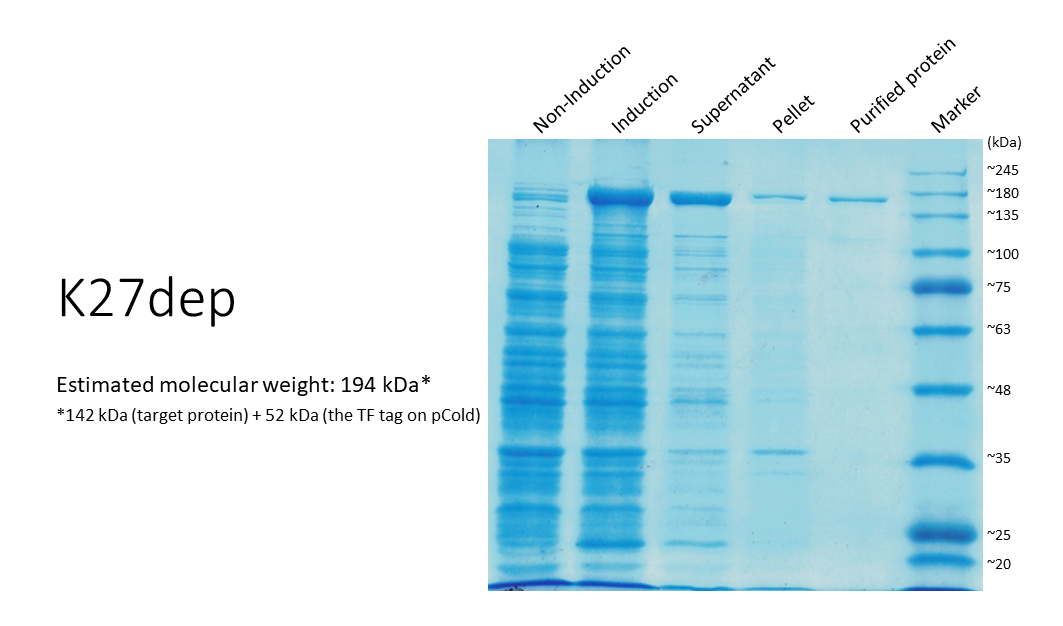


**Fig. S3**

**Fig. S3.** **Coomassie blue staining of SDS-PAGE gels showing the process of protein expression and purification of K7dep (*orf90*), K20dep (*orf93*), and K27dep (*orf91*)**

Each sample (10 µl) was mixed with 10 μl of 2X SDS dye (1:1) and boiled for 5 min. An SDS-PAGE was conducted in 1X running buffer at 20 mA for ~1 h, followed by 40 mA for another ~1 h. Coomassie blue (20% ethanol supplemented) was then applied for staining. The marker used was BLUeye prestained protein ladder (GeneDireX, Taoyuan, Taiwan). The molecular weights were estimated using Expasy Translate (<https://web.expasy.org/translate/>) and Expasy ProtParam (<https://web.expasy.org/protparam/>), provided by the Swiss Institute of Bioinformatics. In addition to the band with estimated molecular weight of K7dep (127 kDa), a band with smaller molecular weight of ~25 kDa was also seen after purification; it is still unknown whether this band is part of K7dep or a non-specific protein. A small amount of proteins with unexpected molecular weights were also present in K20dep purification experiment. K20dep and K27dep expressed in the pCold plasmids that also express trigger factor (TF) chaperone as a soluble tag were further added (52 kDa). Based on the SDS-PAGE gel of K27dep, the molecular weight of the target protein with the TF tag on pCold was smaller (~170 kDa) than expected (194 kDa), which could have resulted from protein cleavage.


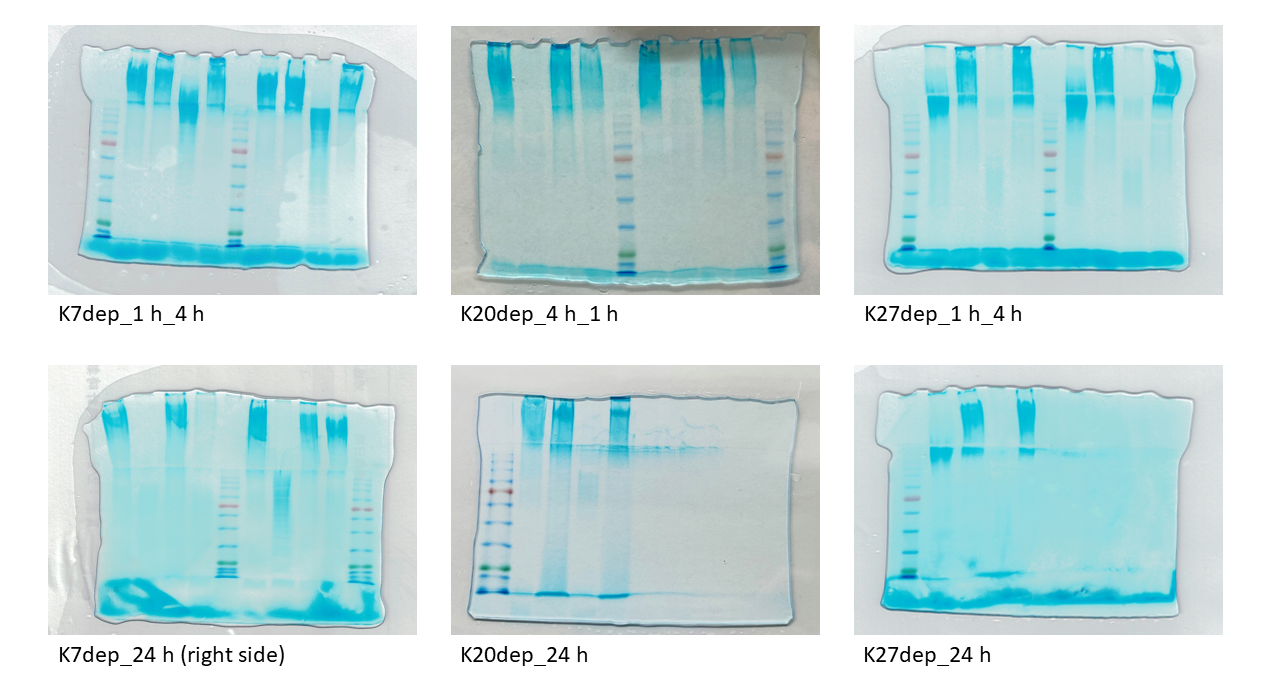


**Fig. S4**

**Fig. S4. Original images of SDS-PAGE gels stained with Alcian blue of purified EPS treated with K7dep, K20dep, and K27dep for 1, 4, or 24 h**

Complete description in the text and Fig. 6.
