# Supplementary material for: Identification of three capsule depolymerases in a bacteriophage infecting Klebsiella pneumoniae capsular types K7, K20, and K27 and therapeutic application
Source: J Biomed Sci. 2023 May 20;30:31. doi: 10.1186/s12929-023-00928-0 (PMC10199534; doi:10.1186/s12929-023-00928-0)
Supplement: Supplementary file 3 — Additional file 3. Methods. Plasmid construction. Expression and purification of putative capsule depolymerases. Coomassie blue staining and quantification of capsule depolymerases. Results. NCBI protein BLAST-Multiple Sequence Alignment viewer [file 12929_2023_928_MOESM3_ESM.pdf]

Additional file 3

Methods

Plasmid construction

To generate N-terminal $(His)_6$-tagged proteins, putative capsule depolymerase genes were inserted into either a pET-28c expression vector (Novagen, Madison, WI, United States) or a pCold TF DNA expression system (Takara, Tokyo, Japan), which can increase protein solubility and production. For *orf90*, a pET-28c expression vector was digested with EcoRI (NEB, Ipswich, MA, United States), blunted with DNA Polymerase I, Large (Klenow) Fragment (NEB), and ligated with the PCR products of *orf90* by T4 ligation system (NEB). For *orf91*, a pCold plasmid and PCR products of *orf91* were digested with XhoI/SacI and ligated with each other. Similar procedures were performed for *orf92* (XhoI/NdeI) and *orf93* (NdeI/HindIII).

The primers used to construct the capsule depolymerase expression plasmids are listed in **Table S2**. The cycling conditions of PCR were 96°C for 3 min, followed by 30 cycles of 96°C for 30 s, 50°C for 15 s, and 72°C for 3 min with KOD Xtreme Hot Start DNA Polymerase (Novagen).

Expression and purification of putative capsule depolymerases

The resulting plasmids were transformed into *E. coli* BL21(DE3) (Invitrogen, Carlsbad, CA, United States) cells. The proteins were expressed by adding IPTG to a final

concentration of 0.1 mM and incubating at 15°C overnight. "Non-induction" and "induction" samples were collected with the same amounts of bacterial cultures. IPTG-induced bacterial culture (20 ml) was centrifugated and resuspended in 10 ml of lysis buffer (300 mM NaCl, 10 mM imidazole, 50 mM NaH_2PO_4). Soluble ("supernatant") and insoluble ("pellet") fractions were sampled after sonication and centrifugation. The His-tagged proteins in the remaining supernatant were purified using nickel beads (GE Healthcare, Uppsala, Sweden) according to the manufacturer's instructions. Briefly, 30 µl of the beads was used to bind proteins at 4°C overnight. The beads were then washed five times, 20 ml/time, with wash buffer (1000 mM NaCl, 20 mM imidazole, 50 mM NaH_2PO_4), and proteins were eluted with 100 µl elution buffer (300 mM NaCl, 100 mM imidazole, 50 mM NaH_2PO_4) and stored in this buffer as "purified protein". For Alcian blue staining or animal experiments that need large amounts of proteins, high volumes of bacterial culture were used for protein expression, and the eluted protein was further concentrated with Microsep Advance Centrifugal Devices with Omega Membrane 100K MWCO (Pall, Port Washington, NY, United States).

Coomassie blue staining and quantification of capsule depolymerases

Purified proteins were analyzed in 10% sodium dodecyl sulfate-polyacrylamide gel electrophoresis (SDS-PAGE) gels stained with 20% ethanol supplemented Coomassie blue (VWR, Radnor, PA, United States). Each sample (non-induction, induction

supernatant, pellet, and purified protein; 10 μl) was mixed with 10 μl of 2X SDS dye (1:1) and boiled for 5 min. SDS-PAGE was conducted in 1X running buffer at 20 mA for ~1 h, followed by 40 mA for another ~1 h, and the gel was stained. The molecular weights of target proteins were estimated using Expasy Translate (https://web.expasy.org/translate/) and Expasy ProtParam (https://web.expasy.org/protparam/), provided by the Swiss Institute of Bioinformatics. BLUeye prestained protein ladder (GeneDireX, Taoyuan, Taiwan) was used to indicate the molecular weights ranging from ~11 to ~245 kDa on SDS-PAGE gels. Concentration of purified proteins was estimated using the Dual-Range™ BCA Protein Assay Kit (Visual Protein, Taipei, Taiwan) based on the manufacturer's instructions.

Results

<u>NCBI protein BLAST-Multiple Sequence Alignment viewer</u>

Protein BLAST results of ORF90, ORF91, ORF92, and ORF93 are available in this order in the following pages which show the alignments with highly conserved (red) and less conserved (blue) amino acid positions by the coloring method of "Conservation" using the Multiple Sequence Alignment viewer.

NCBI Multiple Sequence Alignment Viewer, Version 1.23.1

Alignment

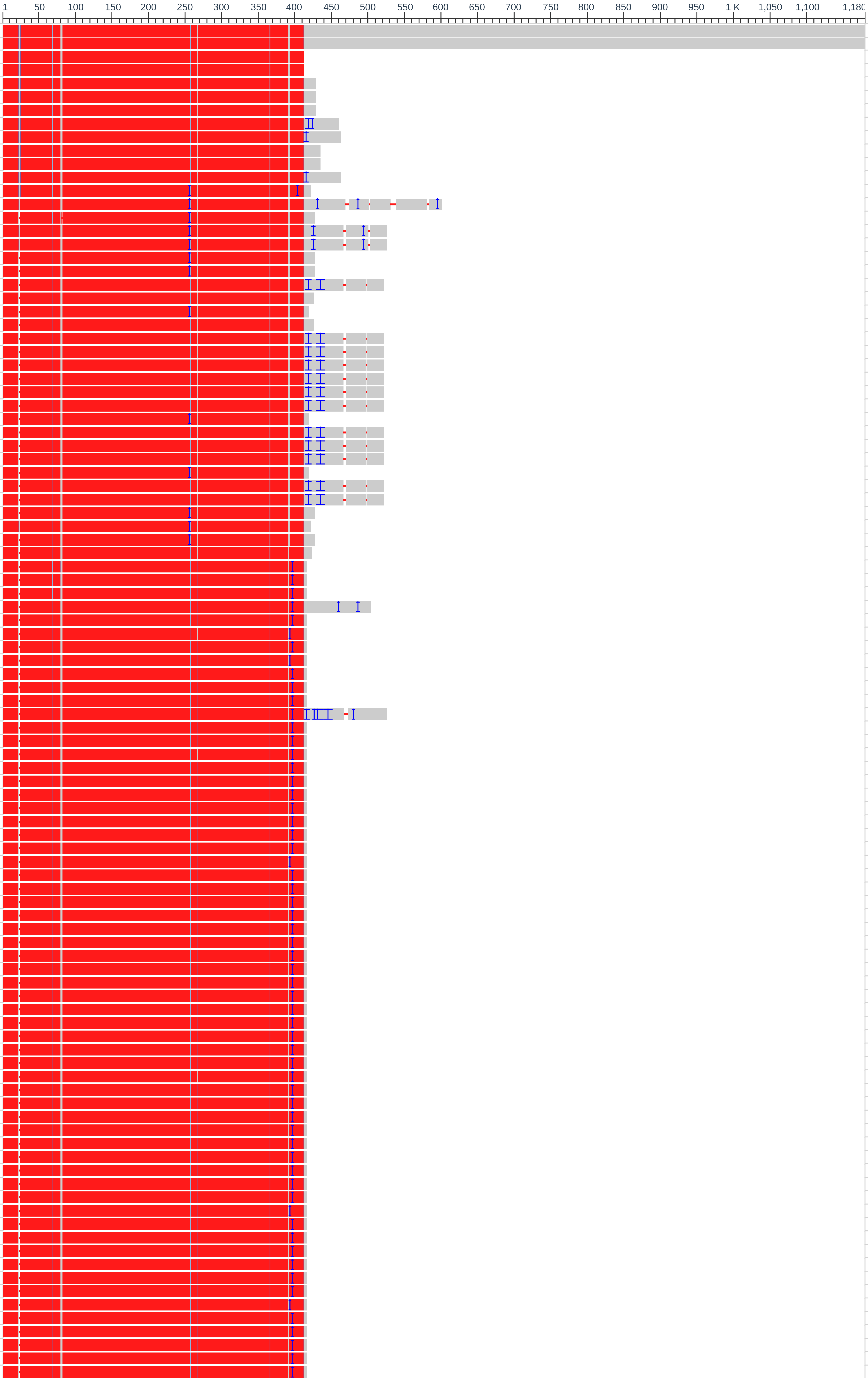

Sequence ID	Start	End	Organism
Query_48455 (+)	1	1,180	
YP_009796159.1 (+)	1	1,180	Klebsiella phage May
YP_009883456.1 (+)	1	412	Klebsiella phage Magnus
YP_009796378.1 (+)	1	412	Klebsiella phage Menlow
UPW36204.1 (+)	1	427	Klebsiella phage K751
YP_009798891.1 (+)	1	427	Klebsiella phage vB_Kp...
UJD04931.1 (+)	1	427	Klebsiella phage PWKp5
YP_008532046.1 (+)	1	471	Klebsiella phage 0507-...
YP_009884728.1 (+)	1	469	Klebsiella phage UPM 2...
CAD5240411.1 (+)	1	434	Klebsiella phage vB_Kq...
CAD5240864.1 (+)	1	434	Klebsiella phage vB_KqM...
YP_009822509.1 (+)	1	469	Escherichia phage vB_...
YP_009616009.1 (+)	1	423	Serratia phage vB-Sru-...
QOI58339.1 (+)	1	582	Salmonella phage pSal...
YP_007236302.1 (+)	1	425	Salmonella phage SKML...
WP_276339511.1 (+)	1	521	Escherichia coli
USL85809.1 (+)	1	521	Enterobacter phage fGh...
YP_009889340.1 (+)	1	425	Salmonella phage P46FS4
YP_003358665.1 (+)	1	425	Shigella phage Ag3
QOE31980.1 (+)	1	535	Salmonella phage ISTP3
QHB41667.1 (+)	1	420	Dickeya phage Ds5CZ
UHS65015.1 (+)	11	427	Shigella phage vB_SboS...
YP_007237482.1 (+)	1	420	Dickeya phage vB-Dso...
QMV34169.1 (+)	1	535	Salmonella phage vB_...
QPI14501.1 (+)	1	535	Salmonella phage GEC...
QEA10161.1 (+)	1	535	Salmonella phage Mata...
UZV39253.1 (+)	1	535	Salmonella phage vB_H...
YP_009101511.1 (+)	1	535	Escherichia phage ECM...
AYJ73683.1 (+)	1	535	Salmonella phage PS5
UGO46943.1 (+)	11	427	Shigella phage vB_Sbo...
QPX73968.1 (+)	1	535	Salmonella phage vB_S...
EHJ9376286.1 (+)	1	535	Salmonella enterica
YP_004327544.1 (+)	1	535	Salmonella phage Vi01
YP_009882836.1 (+)	1	417	Shigella phage MK-13
YP_008770865.1 (+)	1	535	Salmonella phage Mayn...
EJD3358023.1 (+)	1	535	Salmonella enterica
YP_009102965.1 (+)	1	425	Dickeya phage RC-2014
YP_009877005.1 (+)	1	420	Enterobacter phage Es...
AIM51760.1 (+)	1	425	Dickeya phage phiDP23.1
YP_009887919.1 (+)	1	419	Salmonella phage bering
URQ08737.1 (+)	1	412	Salmonella phage BRM ...
EGJ6623211.1 (+)	1	412	Salmonella enterica
WAK45312.1 (+)	1	412	Salmonella phage 3384...
UPU15645.1 (+)	1	505	Salmonella phage STP55
UMO77667.1 (+)	1	412	Salmonella phage PLL1
UHS65529.1 (+)	1	413	Escherichia phage vB_...
QQO88800.1 (+)	1	412	Salmonella phage vB_S...
WP_242244031.1 (+)	1	413	Escherichia coli
UJQ70782.1 (+)	1	412	Salmonella phage selz
UJQ70937.1 (+)	1	412	Salmonella phage selz
YP_004895334.1 (+)	1	412	Salmonella phage SFP10
YP_009887530.1 (+)	1	578	Salmonella phage heyday
YP_009140378.1 (+)	1	412	Salmonella phage Det7
UQS93039.1 (+)	1	412	Salmonella phage BRM ...
WDR21250.1 (+)	1	413	Salmonella phage vB_Se...
YP_009948933.1 (+)	1	412	Salmonella phage Se-B
ARB06439.1 (+)	1	412	Salmonella phage S8
YP_009876468.1 (+)	1	412	Salmonella phage BSP101
YP_009881073.1 (+)	1	412	Salmonella phage Sen...
YP_007008119.1 (+)	1	412	Salmonella phage Sh19
EDW4917992.1 (+)	1	412	Salmonella enterica sub...
YP_009880319.1 (+)	1	412	Escherichia phage EP75
QVW27043.1 (+)	1	413	Escherichia phage vB_...
QPX74345.1 (+)	1	412	Salmonella phage vB_S...
AXF41691.1 (+)	1	412	Salmonella phage vB_Sa...
YP_009030451.1 (+)	1	412	Salmonella phage vB-Sa...
YP_009888728.1 (+)	1	412	Salmonella phage moki
YP_007002809.1 (+)	1	412	Escherichia phage PhaxI
YP_009881274.1 (+)	1	412	Salmonella phage Sen...
YP_009889140.1 (+)	1	412	Salmonella phage maane
CAB5494677.1 (+)	1	412	Salmonella phage Se_AO1
YP_004957867.1 (+)	1	412	Escherichia phage Cba...
YP_009888938.1 (+)	1	412	Salmonella phage perto...
YP_009293364.1 (+)	1	412	Salmonella phage vB-Sa...
WBL99281.1 (+)	1	412	Salmonella phage SPTD1
YP_009889982.1 (+)	1	412	Salmonella phage SE14
YP_009966817.1 (+)	1	412	Salmonella phage Se-J
WDS51544.1 (+)	1	412	Salmonella phage SeF6a
YP_009879584.1 (+)	1	413	Escherichia phage FEC14
YP_009876000.1 (+)	1	412	Salmonella phage ST-...
URQ09037.1 (+)	1	412	Salmonella phage BRM ...
UJQ69934.1 (+)	1	412	Salmonella phage selz
YP_009283777.1 (+)	1	412	Salmonella phage GG32
YP_009879666.1 (+)	1	412	Salmonella phage Mutine
YP_009966642.1 (+)	1	412	Salmonella phage Se-G
UCR92294.1 (+)	1	412	Escherichia phage LPE...
YP_009888531.1 (+)	1	412	Salmonella phage barely
YP_009888327.1 (+)	1	412	Salmonella phage dinky
UTQ72552.1 (+)	1	413	Escherichia phage A221
QPX74959.1 (+)	1	412	Salmonella phage vB_S...
YP_009883090.1 (+)	1	412	Salmonella phage SS9
QIG60374.1 (+)	1	412	Salmonella phage Che...
YP_009881975.1 (+)	1	412	Salmonella phage SeSz-1
YP_009877770.1 (+)	1	412	Salmonella phage SP1
QPX74541.1 (+)	1	412	Citrobacter phage vB_C...
URX65979.1 (+)	1	413	Escherichia phage PC3
WBF04345.1 (+)	1	412	Salmonella phage PST-...
AGF88454.1 (+)	1	412	Salmonella phage FSL ...
YP_009021396.1 (+)	1	412	Salmonella phage vB-Sa...
AGF89194.1 (+)	1	412	Salmonella phage FSL ...
WDR21458.1 (+)	1	412	Salmonella phage vB_Se...

NCBI Multiple Sequence Alignment Viewer, Version 1.23.1

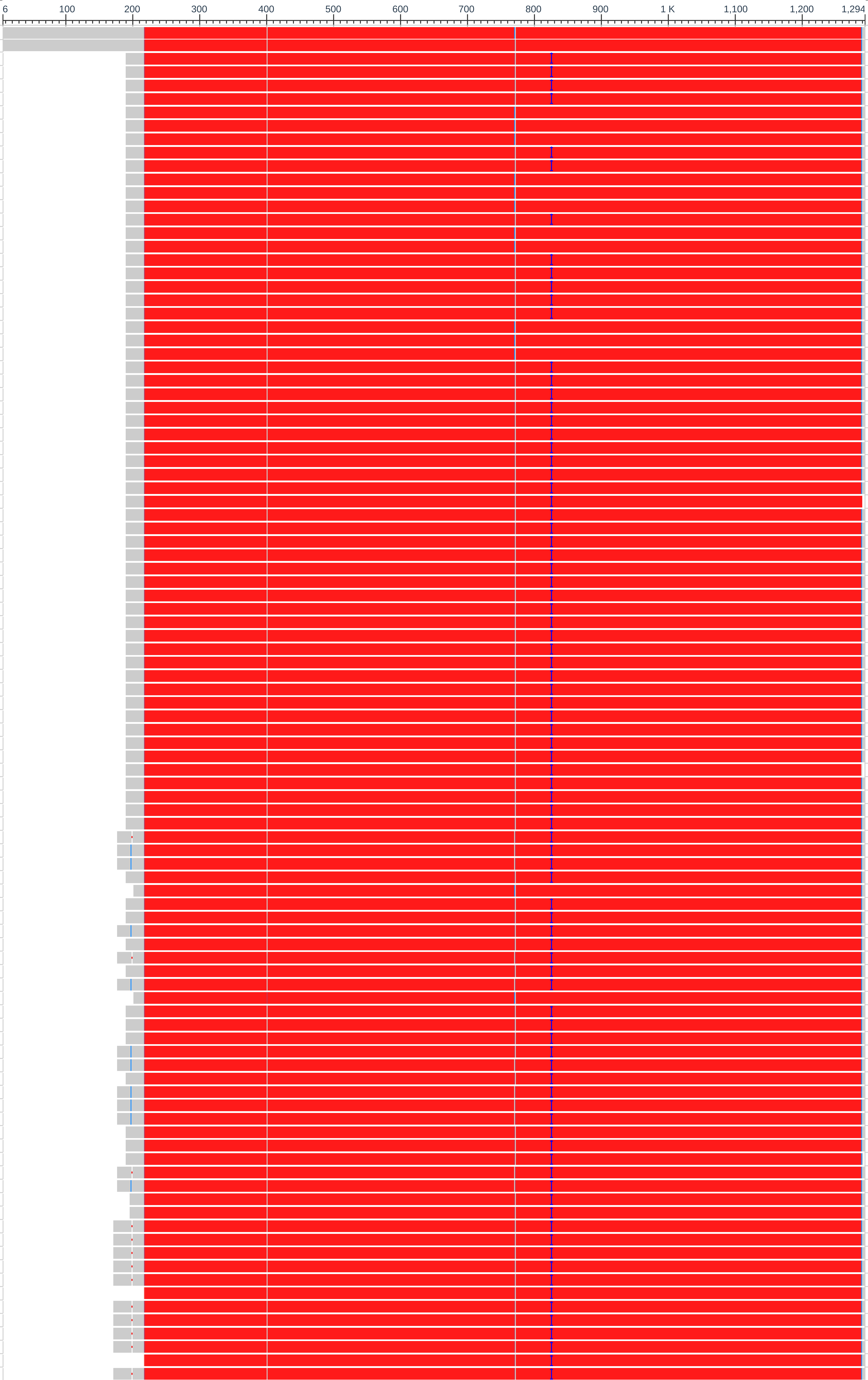

Sequence ID	Start	End	Organism
Query_68372 (+)	6	1,294	
YP_009796160.1 (+)	1	1,288	Klebsiella phage May
MCM5783146.1 (+)	169	1,273	Klebsiella pneumoniae
HBZ7325496.1 (+)	172	1,276	Klebsiella pneumoniae
WP_239594416.1 (+)	169	1,273	Klebsiella pneumoniae
HCC2328278.1 (+)	172	1,276	Klebsiella pneumoniae
WP_032432736.1 (+)	175	1,278	Klebsiella pneumoniae
EWF78718.1 (+)	185	1,288	Klebsiella pneumoniae ...
WP_250833668.1 (+)	113	1,216	Klebsiella pneumoniae
WP_117086717.1 (+)	172	1,276	Klebsiella pneumoniae
HBR5013802.1 (+)	169	1,273	Klebsiella pneumoniae
WP_202560293.1 (+)	175	1,278	Klebsiella pneumoniae
CAE6010988.1 (+)	185	1,288	Klebsiella pneumoniae
WP_039110706.1 (+)	175	1,278	Klebsiella pneumoniae
WP_194429097.1 (+)	169	1,273	Klebsiella pneumoniae
EIV9793011.1 (+)	175	1,278	Klebsiella pneumoniae
SVZ41112.1 (+)	185	1,288	Klebsiella pneumoniae
WP_211662358.1 (+)	169	1,273	Klebsiella pneumoniae
WP_243222088.1 (+)	169	1,273	Klebsiella pneumoniae
WP_117058179.1 (+)	169	1,273	Klebsiella pneumoniae
HBQ9341395.1 (+)	172	1,276	Klebsiella pneumoniae
WP_211786693.1 (+)	169	1,273	Klebsiella pneumoniae
CAE6028260.1 (+)	185	1,288	Klebsiella pneumoniae
WP_217542884.1 (+)	175	1,278	Klebsiella pneumoniae
EIW1548045.1 (+)	175	1,278	Klebsiella pneumoniae
HBR6264915.1 (+)	169	1,273	Klebsiella pneumoniae
HBS5729105.1 (+)	175	1,279	Klebsiella pneumoniae
HBQ6168039.1 (+)	169	1,273	Klebsiella pneumoniae
HBS9781483.1 (+)	169	1,273	Klebsiella pneumoniae
WP_204779816.1 (+)	172	1,276	Klebsiella pneumoniae
WP_188133337.1 (+)	169	1,273	Klebsiella pneumoniae
WP_064362799.1 (+)	169	1,273	Klebsiella grimontii
HBV8052066.1 (+)	169	1,273	Klebsiella pneumoniae
MBD7734763.1 (+)	169	1,273	Klebsiella pneumoniae
EIX9313470.1 (+)	169	1,273	Klebsiella pneumoniae
HBS5706322.1 (+)	172	1,272	Klebsiella pneumoniae
WP_102030388.1 (+)	172	1,276	Klebsiella pneumoniae
MCP6081002.1 (+)	169	1,273	Klebsiella pneumoniae
HBW7898519.1 (+)	169	1,273	Klebsiella pneumoniae
WP_241477096.1 (+)	169	1,273	Klebsiella pneumoniae
CAF2878505.1 (+)	169	1,273	Klebsiella oxytoca
HBU5911795.1 (+)	169	1,273	Klebsiella pneumoniae
MCP6766171.1 (+)	169	1,273	Klebsiella pneumoniae
HBR7982409.1 (+)	172	1,276	Klebsiella pneumoniae
WP_200541202.1 (+)	169	1,273	Klebsiella pneumoniae
WP_064162835.1 (+)	169	1,273	Klebsiella pneumoniae
WP_211603609.1 (+)	169	1,273	Klebsiella pneumoniae
HCM5843957.1 (+)	169	1,273	Klebsiella pneumoniae
WP_129059885.1 (+)	169	1,273	Klebsiella pneumoniae
HBR3569905.1 (+)	172	1,276	Klebsiella pneumoniae
MBK2741127.1 (+)	169	1,273	Klebsiella pneumoniae
HBQ3062887.1 (+)	172	1,276	Klebsiella pneumoniae
WP_273860825.1 (+)	169	1,273	Klebsiella grimontii
WP_186932012.1 (+)	169	1,273	Klebsiella
HBR0870065.1 (+)	169	1,273	Klebsiella pneumoniae
WP_239594369.1 (+)	169	1,270	Klebsiella pneumoniae
WP_274890107.1 (+)	169	1,273	Klebsiella pneumoniae
WP_264970802.1 (+)	172	1,276	Klebsiella variicola
CAF9445906.1 (+)	169	1,273	Klebsiella pneumoniae
WP_109256379.1 (+)	172	1,276	Klebsiella pneumoniae
WP_211677188.1 (+)	254	1,367	Klebsiella pneumoniae
WP_254912817.1 (+)	270	1,383	Klebsiella pneumoniae
WP_227537008.1 (+)	271	1,384	Klebsiella pneumoniae
CAE7274865.1 (+)	172	1,276	Klebsiella pneumoniae
EUC90092.1 (+)	236	1,326	Klebsiella oxytoca OK-1
WP_163585612.1 (+)	172	1,276	Klebsiella pneumoniae
WP_117251216.1 (+)	172	1,276	Klebsiella pneumoniae
SVO18139.1 (+)	269	1,382	Klebsiella pneumoniae
MCJ7282118.1 (+)	172	1,276	Klebsiella pneumoniae
WP_154968374.1 (+)	254	1,367	Klebsiella
WP_217585376.1 (+)	172	1,276	Klebsiella pneumoniae
GKN04887.1 (+)	269	1,382	Klebsiella variicola
RCH14408.1 (+)	236	1,325	Klebsiella pneumoniae
WP_217542923.1 (+)	172	1,276	Klebsiella pneumoniae
CAF2854639.1 (+)	169	1,273	Klebsiella oxytoca
QJO13019.1 (+)	172	1,276	Klebsiella pneumoniae
VCY13181.1 (+)	269	1,382	Klebsiella pneumoniae
WP_274489824.1 (+)	271	1,384	Klebsiella variicola
HBW7671169.1 (+)	169	1,273	Klebsiella pneumoniae
CAH3638288.1 (+)	270	1,383	Klebsiella pneumoniae
WP_227541001.1 (+)	271	1,384	Klebsiella pneumoniae
WP_243637340.1 (+)	271	1,384	Klebsiella pneumoniae
CAF1958164.1 (+)	169	1,273	Klebsiella pneumoniae
WP_224227596.1 (+)	172	1,276	Klebsiella oxytoca
HCB1754683.1 (+)	172	1,274	Klebsiella oxytoca
HCB1751480.1 (+)	244	1,357	Klebsiella oxytoca
WP_259678708.1 (+)	271	1,384	Klebsiella pneumoniae
WP_150395905.1 (+)	175	1,273	Klebsiella pneumoniae
HBS6170707.1 (+)	175	1,273	Klebsiella pneumoniae
QJO53693.1 (+)	113	1,233	Klebsiella pneumoniae
WP_273863206.1 (+)	113	1,233	Klebsiella grimontii
WP_104460793.1 (+)	113	1,233	Klebsiella
EIV7612903.1 (+)	113	1,233	Klebsiella pneumoniae
WP_115698537.1 (+)	113	1,233	Klebsiella pneumoniae
WP_250833718.1 (+)	97	1,174	Klebsiella pneumoniae
WP_124042965.1 (+)	113	1,233	Klebsiella pneumoniae
MBV0597437.1 (+)	113	1,233	Klebsiella pneumoniae
WP_142764690.1 (+)	56	1,176	Klebsiella pneumoniae
HCC2552077.1 (+)	113	1,233	Klebsiella pneumoniae
WP_228296833.1 (+)	97	1,174	Klebsiella pneumoniae
WP_259603040.1 (+)	113	1,233	Klebsiella pneumoniae

NCBI Multiple Sequence Alignment Viewer, Version 1.23.1

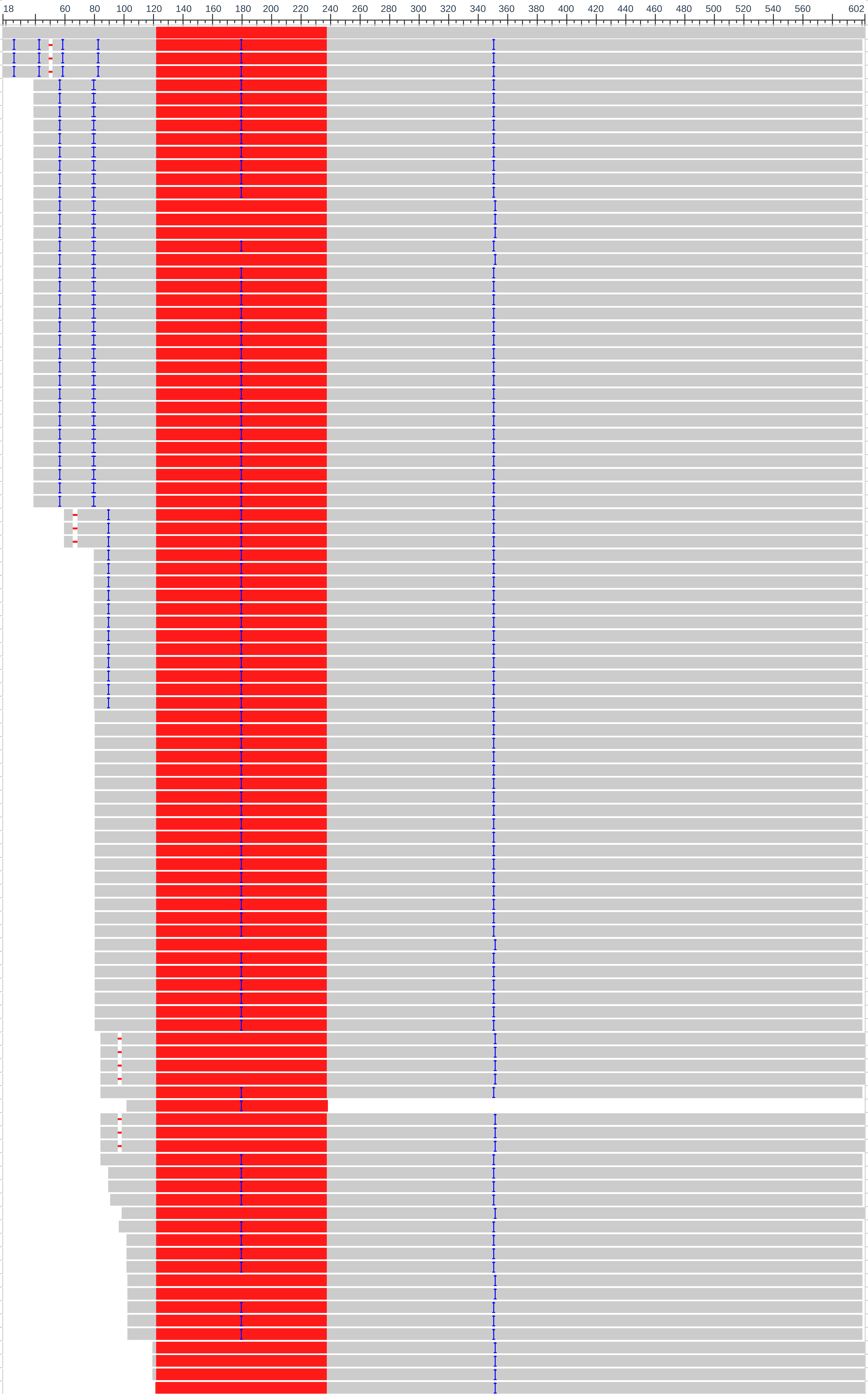

Sequence ID	Start	End	Organism
Query_145325 (+)	18	602	
WP_239719332.1 (+)	46	632	Klebsiella quasipneum...
WP_229301426.1 (+)	46	632	Klebsiella pneumoniae
WP_228727627.1 (+)	46	632	Klebsiella pneumoniae
WP_256332020.1 (+)	236	804	Klebsiella variicola
WP_227112561.1 (+)	235	803	Klebsiella pneumoniae
QMV60554.1 (+)	235	803	Klebsiella pneumoniae
MCL7862806.1 (+)	236	804	Klebsiella pneumoniae
EIW1028138.1 (+)	235	803	Klebsiella pneumoniae
WP_269224712.1 (+)	235	803	Klebsiella pneumoniae
WP_227542476.1 (+)	236	804	Klebsiella pneumoniae
EJM8616797.1 (+)	235	803	Klebsiella pneumoniae
HBR1610390.1 (+)	235	803	Klebsiella pneumoniae
WP_262361508.1 (+)	236	802	Klebsiella pneumoniae
WP_023343148.1 (+)	235	801	Klebsiella pneumoniae
WP_187416778.1 (+)	235	801	Klebsiella pneumoniae
SAR89379.1 (+)	235	803	Klebsiella variicola
WP_228297067.1 (+)	236	802	Klebsiella pneumoniae
CAE7099937.1 (+)	235	803	Klebsiella pneumoniae
WP_233752503.1 (+)	236	804	Klebsiella pneumoniae
HBT0267534.1 (+)	235	803	Klebsiella pneumoniae
WP_262196808.1 (+)	275	843	Klebsiella pneumoniae
WP_229532074.1 (+)	236	804	Klebsiella pneumoniae
MCB7962727.1 (+)	235	803	Klebsiella pneumoniae
WP_227517080.1 (+)	236	804	Klebsiella pneumoniae
WP_022644698.1 (+)	235	803	Klebsiella pneumoniae
MCL3270202.1 (+)	235	803	Klebsiella pneumoniae
WP_258004628.1 (+)	236	804	Klebsiella pneumoniae
UMC65690.1 (+)	234	802	Klebsiella pneumoniae
WP_267695796.1 (+)	236	804	Klebsiella pneumoniae
MCJ5509180.1 (+)	236	804	Klebsiella pneumoniae
EIV6983472.1 (+)	235	803	Klebsiella pneumoniae
WP_246842263.1 (+)	236	804	Klebsiella pneumoniae
EJC6089604.1 (+)	235	803	Klebsiella pneumoniae
WP_227660916.1 (+)	236	804	Klebsiella pneumoniae
WP_159174346.1 (+)	235	803	Klebsiella pneumoniae
WP_258540480.1 (+)	116	656	Klebsiella variicola
WP_233438185.1 (+)	116	656	Klebsiella
WP_258923503.1 (+)	116	656	Klebsiella pneumoniae
VEB96529.1 (+)	132	656	Klebsiella pneumoniae
WP_258961061.1 (+)	132	656	Klebsiella pneumoniae
WP_232692188.1 (+)	132	656	Klebsiella pneumoniae
WP_231545030.1 (+)	132	656	Klebsiella pneumoniae
WP_228260407.1 (+)	172	696	Klebsiella pneumoniae
WP_241772910.1 (+)	31	555	Klebsiella pneumoniae
WP_258932736.1 (+)	172	696	Klebsiella pneumoniae
WP_253192002.1 (+)	132	656	Klebsiella pneumoniae
WP_253248422.1 (+)	132	656	Klebsiella pneumoniae
WP_227636850.1 (+)	172	696	Klebsiella pneumoniae
WP_255152247.1 (+)	132	656	Klebsiella pneumoniae
WP_253195829.1 (+)	132	656	Klebsiella pneumoniae
WP_231545024.1 (+)	134	656	Klebsiella pneumoniae
WP_254894334.1 (+)	75	597	Klebsiella pneumoniae
WP_256924711.1 (+)	134	656	Klebsiella pneumoniae
WP_227661013.1 (+)	131	653	Klebsiella pneumoniae
WP_225371202.1 (+)	131	653	Klebsiella pneumoniae
WP_246844651.1 (+)	134	656	Klebsiella pneumoniae
WP_228725463.1 (+)	134	656	Klebsiella pneumoniae
WP_257016173.1 (+)	36	558	Klebsiella pneumoniae
WP_253884059.1 (+)	134	656	Klebsiella pneumoniae
WP_258551524.1 (+)	134	656	Klebsiella pneumoniae
WP_258958616.1 (+)	134	656	Klebsiella pneumoniae
WP_259280123.1 (+)	134	656	Klebsiella pneumoniae
WP_227142835.1 (+)	134	656	Klebsiella pneumoniae
WP_262387461.1 (+)	134	656	Klebsiella pneumoniae
WP_256330484.1 (+)	131	653	Klebsiella pneumoniae
WP_253249572.1 (+)	134	656	Klebsiella pneumoniae
WP_227818742.1 (+)	134	656	Klebsiella pneumoniae
WP_227649543.1 (+)	134	654	Klebsiella pneumoniae
WP_259271858.1 (+)	134	656	Klebsiella pneumoniae
WP_245201903.1 (+)	25	547	Klebsiella pneumoniae
WP_227640195.1 (+)	131	653	Klebsiella pneumoniae
WP_258963546.1 (+)	131	653	Klebsiella pneumoniae
WP_255037936.1 (+)	84	606	Klebsiella pneumoniae
WP_227602126.1 (+)	134	656	Klebsiella pneumoniae
WP_258785776.1 (+)	219	734	Klebsiella pneumoniae
HBR2602864.1 (+)	220	735	Klebsiella pneumoniae
WP_228722654.1 (+)	219	734	Klebsiella pneumoniae
WP_181502371.1 (+)	220	735	Klebsiella pneumoniae
VGJ37168.1 (+)	166	684	Klebsiella pneumoniae
VGJ37168.1 (+)	1	138	Klebsiella pneumoniae
WP_242634505.1 (+)	219	734	Klebsiella pneumoniae
WP_153938069.1 (+)	220	735	Klebsiella
WP_255039260.1 (+)	220	735	Klebsiella pneumoniae
WP_228261524.1 (+)	16	534	Klebsiella pneumoniae
MCU0163376.1 (+)	1	514	Klebsiella pneumoniae
WP_267671115.1 (+)	1	514	Klebsiella pneumoniae
WP_256924583.1 (+)	338	850	Klebsiella pneumoniae
UPW36206.1 (+)	282	786	Klebsiella phage K751
MCP2473150.1 (+)	1	507	Klebsiella pneumoniae
STW17919.1 (+)	1	502	Klebsiella pneumoniae
SVT81259.1 (+)	1	502	Klebsiella pneumoniae
SLY16057.1 (+)	1	502	Klebsiella pneumoniae
WP_161737648.1 (+)	1	499	Klebsiella pneumoniae
MCG0522703.1 (+)	1	499	Klebsiella pneumoniae
MBP0696222.1 (+)	1	501	Klebsiella pneumoniae
WP_080851607.1 (+)	1	501	Klebsiella pneumoniae
HCB1291290.1 (+)	1	501	Klebsiella pneumoniae
WP_159174406.1 (+)	1	484	Klebsiella pneumoniae
MBK2378951.1 (+)	1	484	Klebsiella pneumoniae
HCM3837847.1 (+)	1	484	Klebsiella quasipneum...
WP_227648749.1 (+)	1	482	Klebsiella pneumoniae

NCBI Multiple Sequence Alignment Viewer, Version 1.23.1

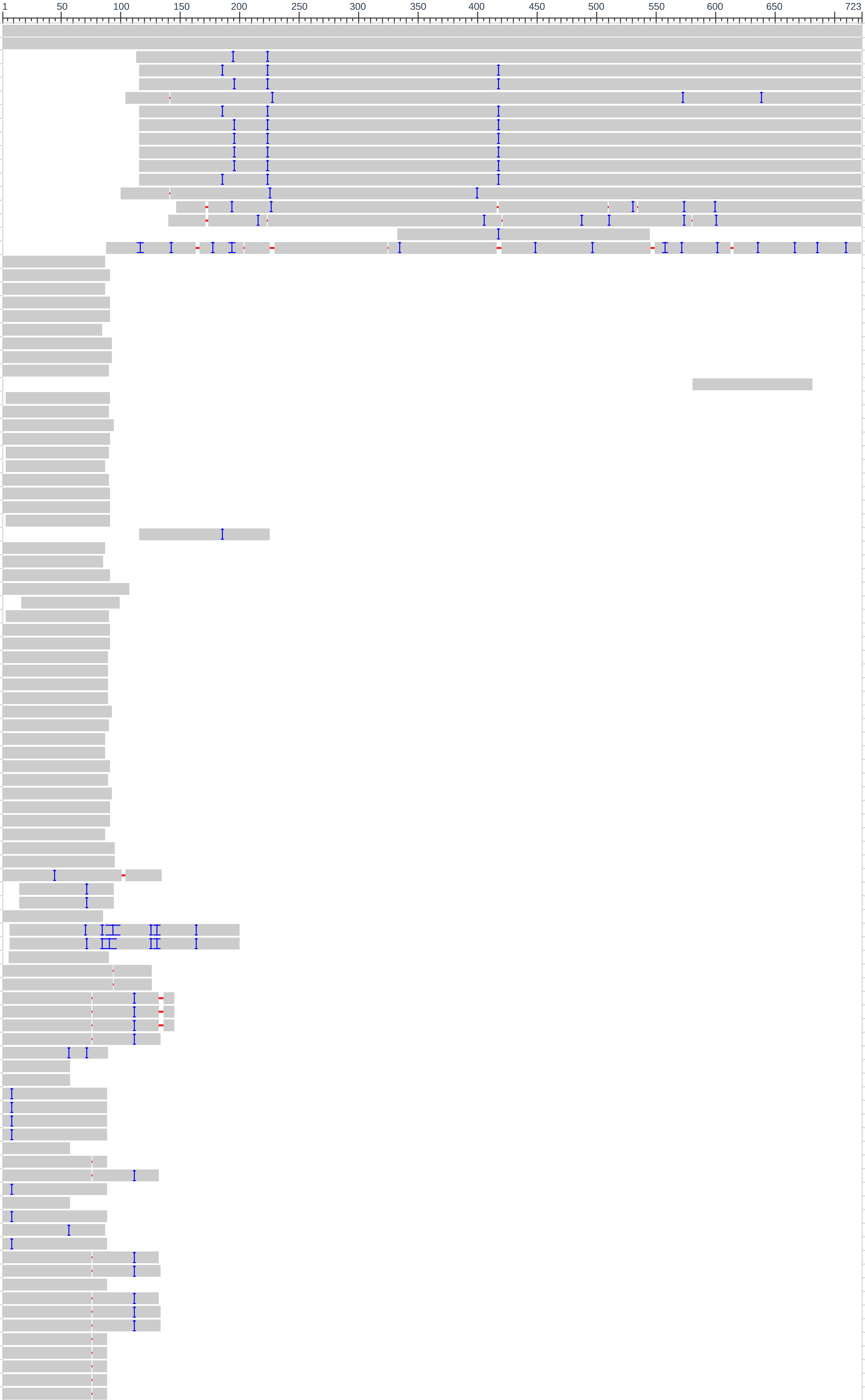

Sequence ID	Start	End	Organism
Query_182876 (+)	1	723	
UPW36008.1 (+)	1	723	Klebsiella phage K751
UEW68236.1 (+)	205	814	Klebsiella phage vB_Kp...
WBU87607.1 (+)	294	902	Klebsiella phage ZCKP3
YP_009786888.1 (+)	294	902	Klebsiella phage vB_Kp...
QYC97042.1 (+)	18	635	Klebsiella phage IME184
UEP19662.1 (+)	294	902	Klebsiella phage vB_Kp...
YP_009801472.1 (+)	294	902	Klebsiella phage KP32_...
YP_009215498.1 (+)	294	902	Klebsiella phage vB_Kp...
YP_004678762.1 (+)	294	902	Escherichia phage K30
WAW44464.1 (+)	294	902	Klebsiella phage Kp_G...
WAW44388.1 (+)	294	902	Klebsiella phage Kp_G...
WP_064357333.1 (+)	159	780	Raoultella ornithinolytica
QKJ86813.1 (+)	100	670	Erwiniaceae bacterium ...
WP_202809850.1 (+)	80	660	Serratia fonticola
WP_236494618.1 (+)	1	213	Escherichia coli
WP_132927127.1 (+)	15	657	Sodalis ligni
YP_009798897.1 (+)	1	86	Klebsiella phage vB_Kp...
CAD5240429.1 (+)	1	90	Klebsiella phage vB_Kq...
UJD04937.1 (+)	1	86	Klebsiella phage PWKp5
YP_008532049.1 (+)	46	135	Klebsiella phage 0507-...
YP_009796163.1 (+)	1	90	Klebsiella phage May
YP_009822516.1 (+)	1	83	Escherichia phage vB_...
YP_009883462.1 (+)	1	91	Klebsiella phage Magnus
YP_009796383.1 (+)	1	91	Klebsiella phage Menlow
YP_009884734.1 (+)	1	89	Klebsiella phage UPM 2...
WP_236494671.1 (+)	2	101	Escherichia coli
HBT0444912.1 (+)	3	89	Klebsiella pneumoniae
DAG71597.1 (+)	1	89	Caudoviricetes sp.
QOE32392.1 (+)	1	93	Klebsiella phage Muenster
UYL05426.1 (+)	1	90	Klebsiella phage KP13-7
BBK09261.1 (+)	2	87	Klebsiella phage 05F01
UYL05422.1 (+)	3	85	Klebsiella phage KP13-7
HBU4529552.1 (+)	1	89	Klebsiella pneumoniae
DAL41233.1 (+)	1	90	Caudoviricetes sp.
DAK05611.1 (+)	33	122	Caudoviricetes sp.
UYL04389.1 (+)	3	89	Klebsiella phage KP13-16
WP_236494615.1 (+)	243	352	Escherichia coli
YP_009153197.1 (+)	1	86	Klebsiella phage K64-1
YP_009616015.1 (+)	1	84	Serratia phage vB-Sru-...
DAE75140.1 (+)	1	90	Caudoviricetes sp.
YP_009616013.1 (+)	1	106	Serratia phage vB-Sru-...
WP_222706807.1 (+)	2	83	Lacisediminihabitans pro...
DAY73613.1 (+)	3	88	Caudoviricetes sp.
YP_008532048.1 (+)	1	90	Klebsiella phage 0507-...
DAH87420.1 (+)	20	109	Caudoviricetes sp.
YP_009883460.1 (+)	1	88	Klebsiella phage Magnus
YP_009796161.1 (+)	1	88	Klebsiella phage May
YP_009884732.1 (+)	1	88	Klebsiella phage UPM 2...
YP_009796381.1 (+)	1	88	Klebsiella phage Menlow
DAE64591.1 (+)	17	107	Caudoviricetes sp.
YP_009822514.1 (+)	1	89	Escherichia phage vB_...
UPW36207.1 (+)	1	86	Klebsiella phage K751
URG13722.1 (+)	1	86	Klebsiella phage T751
UYL04807.1 (+)	1	90	Klebsiella phage KP13-26
QOE32399.1 (+)	1	88	Klebsiella phage Muenster
UOX39352.1 (+)	1	91	Klebsiella phage GADU21
UVX30945.1 (+)	1	90	Klebsiella phage VLCpi...
UVX31118.1 (+)	1	90	Klebsiella phage VLCpi...
WAK45169.1 (+)	1	86	Klebsiella phage VB_K...
CAD5240880.1 (+)	1	94	Klebsiella phage vB_KqM...
YP_010114883.1 (+)	1	94	Klebsiella phage vB_Kp...
UYL05423.1 (+)	1	129	Klebsiella phage KP13-7
WP_142468683.1 (+)	4	83	Klebsiella pasteurii
WP_189094993.1 (+)	4	83	Klebsiella grimontii
UZN24474.1 (+)	1	84	Klebsiella phage pKP-B...
YP_009153200.1 (+)	7	231	Klebsiella phage K64-1
YP_007007685.1 (+)	7	231	Klebsiella phage vB_Kl...
YP_010563516.1 (+)	5	88	Klebsiella phage vB_Kp...
ASZ78809.1 (+)	1	123	Serratia phage 2050H1
YP_007349017.1 (+)	1	123	Serratia phage phiMAM1
AXY85150.1 (+)	1	137	Salmonella phage Mooltan
AYJ73680.1 (+)	1	137	Salmonella phage PS5
YP_008771659.1 (+)	1	137	Salmonella phage Marshall
YP_009877767.1 (+)	1	130	Salmonella phage SP1
QOE32390.1 (+)	1	90	Klebsiella phage Muenster
EAS3162924.1 (+)	4	59	Salmonella enterica
QPX73971.1 (+)	1	56	Salmonella phage vB_S...
YP_009984888.1 (+)	1	87	Escherichia phage vB_...
WAX24755.1 (+)	1	87	Escherichia phage vB_...
YP_009803406.1 (+)	1	87	Klebsiella phage ZCKP1
HCJ8179831.1 (+)	1	87	Escherichia coli
YP_009101515.1 (+)	1	56	Escherichia phage ECM...
QMV34172.1 (+)	1	85	Salmonella phage vB_...
YP_008770868.1 (+)	1	129	Salmonella phage Mayn...
HCJ8320404.1 (+)	1	87	Escherichia coli
EHJ9376289.1 (+)	1	56	Salmonella enterica
YP_009986343.1 (+)	1	87	Escherichia phage niez...
YP_007007681.1 (+)	1	87	Klebsiella phage vB_Kl...
HBC8516778.1 (+)	1	87	Escherichia coli
QOE31977.1 (+)	1	129	Salmonella phage ISTP3
YP_009220808.1 (+)	1	130	Salmonella phage 38
QXV83763.1 (+)	1	86	Escherichia phage Paul...
WBL99286.1 (+)	1	129	Salmonella phage SPTD1
YP_009889142.1 (+)	1	130	Salmonella phage maane
YP_009881782.1 (+)	1	130	Salmonella phage SeSz-1
QPI15382.1 (+)	1	85	Salmonella phage GEC...
WES09879.1 (+)	1	85	Salmonella phage SWJ...
YP_009888941.1 (+)	1	85	Salmonella phage perto...
YP_009889984.1 (+)	1	85	Salmonella phage SE14
URQ09040.1 (+)	1	85	Salmonella phage BRM ...
